# A Discovery Resource of Rare Copy Number Variations in Individuals with Autism Spectrum Disorder

**DOI:** 10.1534/g3.112.004689

**Published:** 2012-12-01

**Authors:** Aparna Prasad, Daniele Merico, Bhooma Thiruvahindrapuram, John Wei, Anath C. Lionel, Daisuke Sato, Jessica Rickaby, Chao Lu, Peter Szatmari, Wendy Roberts, Bridget A. Fernandez, Christian R. Marshall, Eli Hatchwell, Peggy S. Eis, Stephen W. Scherer

**Affiliations:** * The Centre for Applied Genomics, Program in Genetics and Genome Biology, The Hospital for Sick Children, Toronto M5G 1L7, Canada; † Department of Molecular Genetics, University of Toronto, Toronto M5G 1L7, Canada; ‡ Offord Centre for Child Studies, Department of Psychiatry and Behavioural Neurosciences McMaster University, Hamilton L8P 3B6, Canada; § Autism Research Unit, The Hospital for Sick Children, Toronto M5G 1X8, Canada; ** Disciplines of Genetics and Medicine, Memorial University of Newfoundland, St. John’s, Newfoundland A1B 3V6, Canada; †† McLaughlin Centre, University of Toronto, Toronto M5G 1L7, Canada; ‡‡ Population Diagnostics, Inc., Melville, New York 11747

**Keywords:** rare variants, gene copy number, chromosomal abnormalities, cytogenetics, molecular pathways

## Abstract

The identification of rare inherited and *de novo* copy number variations (CNVs) in human subjects has proven a productive approach to highlight risk genes for autism spectrum disorder (ASD). A variety of microarrays are available to detect CNVs, including single-nucleotide polymorphism (SNP) arrays and comparative genomic hybridization (CGH) arrays. Here, we examine a cohort of 696 unrelated ASD cases using a high-resolution one-million feature CGH microarray, the majority of which were previously genotyped with SNP arrays. Our objective was to discover new CNVs in ASD cases that were not detected by SNP microarray analysis and to delineate novel ASD risk loci via combined analysis of CGH and SNP array data sets on the ASD cohort and CGH data on an additional 1000 control samples. Of the 615 ASD cases analyzed on both SNP and CGH arrays, we found that 13,572 of 21,346 (64%) of the CNVs were exclusively detected by the CGH array. Several of the CGH-specific CNVs are rare in population frequency and impact previously reported ASD genes (*e.g.*, *NRXN1*, *GRM8*, *DPYD*), as well as novel ASD candidate genes (*e.g.*, *CIB2*, *DAPP1*, *SAE1*), and all were inherited except for a *de novo* CNV in the *GPHN* gene. A functional enrichment test of gene-sets in ASD cases over controls revealed nucleotide metabolism as a potential novel pathway involved in ASD, which includes several candidate genes for follow-up (*e.g.*, *DPYD*, *UPB1*, *UPP1*, *TYMP*). Finally, this extensively phenotyped and genotyped ASD clinical cohort serves as an invaluable resource for the next step of genome sequencing for complete genetic variation detection.

Rare inherited and *de novo* copy number variations (CNVs) contribute to the genetic vulnerability in autism spectrum disorder (ASD) in as many as 5–10% of idiopathic cases examined (Devlin and Scherer 2012). Smaller intragenic CNVs (often called indels) can be involved, or CNVs can encompass an entire gene, and in some cases they can affect several genes as part of a genomic disorder (Lee and Scherer 2010). Screening for CNVs using microarrays has proven to be a rapid method to identify both large and small genomic imbalances associated with ASD susceptibility (Jacquemont *et al.* 2006; Sebat *et al.* 2007; Autism Genome Project Consortium 2007; Christian *et al.* 2008; Marshall *et al.* 2008; Pinto *et al.* 2010; Shen *et al.* 2010; Sanders *et al.* 2011).

A trend emerging from recent investigations in autism genetics is that significant heterogeneity and complexity exists. However, some highly penetrant risk genes (*e.g.*, the *SHANK*, *NRXN*, and *NLGN* family members) and CNV loci (*e.g.*, 1q21.1, 15q13.3, and 16p11.2) are now known (Devlin and Scherer 2012). Moreover, there has been some progress in identifying multiple mutations in single individuals (Schaaf *et al.* 2011), suggesting possible multigenic threshold models for ASD (Cook and Scherer 2008). Scientific designs enabling the discovery of a comprehensive set of genetic variants will be required to fully assess the genetic burden in ASD.

CNV detection in case and control cohorts have been performed using several different microarray platforms, including those utilizing single-nucleotide polymorphisms (SNPs) and others using comparative genomic hybridization (CGH) arrays (Redon *et al.* 2006; Graubert *et al.* 2007; Christian *et al.* 2008; Conrad *et al.* 2010; Pinto *et al.* 2011). There are advantages to each microarray platform. SNP-based microarray platforms are typically cost-effective and have the potential to analyze the genomic DNA sample at both the SNP genotype and copy number level, whereas CGH arrays are often used in a clinical diagnostic setting because of the better signal-to-noise-ratio achieved in comparison to SNP arrays (Shinawi and Cheung 2008). Multiple algorithms, some specific to certain array types, are available to detect CNVs, and they can vary considerably in the number of CNV calls made even for an identical array experiment. In recent comprehensive studies, authors have provided performance assessments of CNV detection platforms and methods by examining control DNA samples (Lai *et al.* 2005; Curtis *et al.* 2009; Hester *et al.* 2009; Winchester *et al.* 2009; Pinto *et al.* 2011). The consensus is that using multiple microarray technologies and prediction algorithms increases CNV detection rates.

In this study, we used a high-resolution Agilent CGH array comprising 1 million (1M) probes to assay for CNVs in a Canadian cohort of 696 unrelated ASD cases, 615 of which were genotyped previously on SNP arrays, including Illumina 1M single/duo (Pinto *et al.* 2010), Affymetrix 500K (Marshall *et al.* 2008), Affymetrix 6.0 (Lionel *et al.* 2011; and A. C. Lionel, unpublished data), and Illumina Omni 2.5M (A. C. Lionel, unpublished data) arrays. Our high-quality data, interpreted for the first time with CNV control data generated on 1000 individuals using the same 1M CGH array, enabled detection of numerous rare CNVs in each ASD sample examined and identified new potential ASD risk genes. These data allowed us to significantly expand the profile of genetic variants that are potentially causative of ASD and to identify novel molecular pathways affecting ASD vulnerability in this cohort.

## Materials and Methods

### DNA samples and CNV analysis

#### ASD case cohort:

The array CGH component of this study included 696 unrelated ASD cases, 39 affected siblings, and 42 parents (17 complete trios) that passed array quality control. All the ASD cases met the criteria for autism on one or both diagnostic measures – Autism Diagnostic Interview-Revised training and Autism Diagnostic Observation Schedule training. The samples came from three Canadian sites: Hospital for Sick Children in Toronto, Ontario; McMaster University, Hamilton, Ontario; and Memorial University of Newfoundland, St. John’s, Newfoundland. For the 696 unrelated ASD cases (571 males and 125 females), CGH experiments were performed on genomic DNA derived from blood for 354 cases, lymphoblastoid cell line DNA was used for 340 cases, and in two instances saliva was the DNA source used.

The SNP microarray component of the study used for comparison included 615 unrelated ASD cases, and the data from 433 of these experiments have been published (Marshall *et al.* 2008; Pinto *et al.* 2010). The CNVs detected in these samples were found using previously described stringent calling criteria defined for two different computer algorithms, which yields reliable CNV calls that can be experimentally validated >95% of the time (Redon *et al.* 2006; Marshall *et al.* 2008; Conrad *et al.* 2010; Pinto *et al.* 2010).

#### Control cohort:

A control cohort of 1000 DNA samples from reportedly healthy donors (502 males and 498 females) were acquired from BioServe (Beltsville, MD) and are part of a collection of >12,000 control samples originally banked by Genomics Collaborative, Inc. (acquired by BioServe in 2007) for the purpose of large-scale genomic studies (Ardlie *et al.* 2002). Donors were consented and deidentified via a protocol approved by the institutional review board. The control DNA samples were derived from apparently healthy white donors older than 45 years of age. Health history information, documented at the time of consent, was used to select the samples based on the following attributes: body mass index between 15 and 35, blood glucose level <125 mg/dL, total cholesterol level between 100 and 300, systolic blood pressure between 100 and 150, and no major diseases (*e.g.*, cancer and neurodegenerative diseases) or psychiatric disorders (*e.g.*, alcoholism, mental illness, and depression). The control cohort DNA samples (also called PDx controls) used in this study are proprietary and currently biobanked at Population Diagnostics, Inc. (Melville, NY) for future microarray, sequencing and genotyping validation experiments.

### Array CGH

The ASD test and reference samples were labeled with Cy5 and Cy3, respectively using Invitrogen BioPrime CGH labeling kit (Invitrogen, Carlsbad, CA). A pool of 50 sex-matched Caucasian control samples was used as a reference. The PDx control test and reference samples were labeled with Cy3 and Cy5, respectively and one sex-matched sample was used as a reference. All samples have been run on Agilent 1M CGH array according to manufacturer’s protocol (Agilent Technologies, Santa Clara, CA). The Agilent 1M CGH array consists of a total of 974,016 probes providing relatively uniform whole genome coverage. The arrays were scanned using the Agilent microarray scanner, and data were extracted using Feature extraction software version 10.5.1.1. The array experiments for ASD cases and PDx controls were run at The Centre for Applied Genomics (Toronto, Canada) and in the service laboratory of Oxford Gene Technology (Oxford, UK), respectively.

### CNV detection and analysis

All array CGH data (both ASD cases and PDx controls) were analyzed in precisely the same manner using two programs—DNA Analytics v.4.0.85 (Agilent Technologies, Santa Clara, CA) and DNAcopy (Venkatraman and Olshen 2007)—to obtain high-confidence calls. At least five consecutive probe sets were used to call a CNV. For DNA Analytics, the aberration algorithm of Aberration Detection Method-2 was used with a threshold of 6.0, a minimum absolute average log2 ratio per region of 0.25, and maximum number of aberrant regions of 10,000 was used to identify all aberrant intervals. A nested filter of two was applied with subsequent removal of nested child calls using a custom script and retaining only the parent aberrations.

The other algorithm used to call CNVs was DNAcopy, from R Bioconductor package, which is a circular binary segmentation algorithm. The default settings and a log2 ratio cutoff of −0.41 and 0.32 for loss and gain, respectively, was used to call CNVs. Any segment with absolute median log2 ratio to median absolute deviation value less than 2 was filtered out. The CNVs detected by DNA Analytics and DNAcopy for each individual were merged using outer probe boundaries. As in our previous SNP microarray studies (Marshall *et al.* 2008; Pinto *et al.* 2010; Lionel *et al.* 2011), we defined a CNV as being ‘stringent’ if it was detected by both algorithms at the sample level. The stringent dataset was utilized for novel rare CNV discovery.

### Quality control

Experiments with poor derivative log ratio spread (DLRs > 0.3) were discarded. Any sample that had an excessive number of CNVs detected using either algorithm, measured by mean plus 3 SDs, was identified as an outlier and removed from further analysis. Any experiment that was a gender-mismatch was removed, and we excluded CNVs that were within centromere proximal cytobands. Twenty ASD cases that had CNVs larger than 5 Mb in size were removed from further downstream analyses of overlap with SNP microarrays, global rare CNV burden analysis and gene-set association tests.

### Rare ASD CNVs

Stringent CNV calls were classified as rare based on three separate stepwise comparisons with control datasets. First, a subset of stringent CNVs found at a frequency <0.5% for the total sample set (including 676 ASD cases and 1000 PDx controls) was compiled. Second, CNV data from an additional 4139 in-house controls were used to filter CNVs found at ≥0.1% frequency and where 50% by length overlapped with CNVs in the in-house controls. The additional controls consisted of 1782 subjects from the Study on Addiction: Genetics and Environment [SAGE (Bierut *et al.* 2010)], a total of 1234 unrelated controls from the Ottawa Heart Institute study (Stewart *et al.* 2009), and 1123 European controls from the PopGen study (Krawczak *et al.* 2006). The SAGE controls were genotyped with Illumina Human 1M-single BeadChip arrays and a subset of stringent CNVs detected by both iPattern (Pinto *et al.* 2010) and QuantiSNP (Colella *et al.* 2007) were used. The Ottawa Heart Institute and PopGen controls were genotyped with Affymetrix Genome-Wide Human SNP 6.0 arrays and the stringent subset consisted of regions that were detected by at least two of the three different CNV calling algorithms, Birdsuite (Korn *et al.* 2008), iPattern, and Affymetrix Genotyping Console. Third, the list of CNVs that overlapped 50% or more by length with known polymorphic regions in the genome (Conrad *et al.* 2010; McCarroll *et al.* 2008) were excluded. The final list of rare CNVs consisted of 1,884 CNVs in ASD cases and 2,299 CNVs in PDx controls.

### CNV overlap With SNP microarrays to identify novel ASD CNVs

For each sample for which CNV calls from SNP microarrays were available, stringent CNVs detected using the Agilent 1M array were overlapped with the stringent CNVs detected by corresponding SNP microarray experiments (Marshall *et al.* 2008; Pinto *et al.* 2010; A.C. Lionel, unpublished data). The CNVs from the SNP arrays were filtered to include only the regions with five probes or more. The CNVs were considered to be novel when 50% or more by length of the detected call was unique to a platform.

### Experimental CNV validation

The experimental validation of novel CNV calls was carried out using SYBR-Green-based (Stratagene) real-time quantitative polymerase chain reaction (qPCR) method by at least two independent PCR assays. Each assay was conducted in triplicate, with one set of primers corresponding to the region of interest and the other mapping to a control region on *FOXP2* at 7q31.1 (serving as a negative diploid control). The ratio between the test and control regions were then determined using standard curve method, and a fold-change less than 0.7 was confirmed as deletion and greater than 1.3 was confirmed as duplication. The parents and siblings were also tested for inheritance and segregation of CNVs, respectively.

### Selection of European ASD cases

Of the 676 unrelated ASD cases that passed QC, 615 of them had SNP microarray data available [Affymetrix Genome-Wide Human SNP Array 6.0, Affymetrix GeneChip Human Mapping 500K Array Set (Commercial and Early Access), Illumina Human1M and Human1M-Duo, and Illumina HumanOmni2.5-4 BeadChip]. We combined this subset of samples with the HapMap3 data set (International HapMap Consortium 2010), which includes individuals of different ethnicity (*e.g.*, 183 Utah residents with Northern and Western European ancestry, 91 Toscani from Italy, 89 Han Chinese from Beijing, 181 Yorubans from Nigeria). For the ancestry analysis, only a subset of ∼30,000 autosomal, non-MHC SNPs that were common to all the platforms were used. We analyzed this data set using multidimensional scaling (MDS) as implemented in PLINK (Purcell *et al.* 2007) to identify a putative group of European ASD cases. To avoid confounding ancestry issues, we only used European ASD cases for global rare CNV burden analyses and gene-set association tests.

### Global rare CNV burden analyses

CNVs mapping to genes were considered for burden analysis whenever at least one exon was encompassed by or impacted by the CNV. For each subject, we calculated the number of rare CNVs, the log10 of the sum of rare CNV sizes, and the total number of genes harboring a rare CNV. CNV burden differences were assessed by comparing the distribution of these statistics between case and control subjects. Specifically, differences in distribution over case and control subjects were tested using the nonparametric Wilcoxon exact test (as implemented in the R package exactRankTests), whereas the magnitude of the difference was assessed looking at the ratio of case and control means. CNV burden differences were assessed for all rare variants together, as well as for deletions and duplications only.

### Gene-set association test

Gene-sets representing pathways, functional annotations and protein domains were tested if they were more frequently affected by rare CNVs in ASD cases compared with controls using the Fisher exact test [FET (Pinto *et al.* 2010)]. Gene-sets were compiled from Gene Ontology annotations (downloaded from NCBI in April 2011), pathway databases (KEGG, Reactome, bioCarta, NCI in March 2011), and protein domains (PFAM, March 2011). We only tested gene-sets with member genes numbering between 25 and 750: 2456 in total, 1939 from Gene Ontology, 414 from pathways, and 103 from PFAM domains. We found that small gene-sets decrease the statistical power of the analysis, whereas larger gene-sets tend to have a very broad biological scope without much useful biological meaning. For each gene-set, we built a contingency matrix with subjects as sampling units. Subjects were categorized as (1) cases or controls and (2) having at least one gene-set harboring a rare CNV or not. On the basis of this contingency table, we tested higher prevalence of rare CNVs in autism probands *vs.* controls using a one-tailed FET. Any subject in which a rare CNV affected more than 20 genes was not considered for the gene-set analysis because these individuals may have a broader set of gene functions perturbed by rare variants, which may consequently reduce the quality of gene-set analysis results. The FET nominal *p* value was corrected for multiple tests using a case/control class permutation procedure to estimate an empirical false discovery rate (FDR). We performed 5000 permutations of case-control labels and for each permutation we tested gene-sets following exactly the same procedure. The *p* values were ranked from lowest (most significant) to highest (least significant) and for each *p* value we computed the empirical FDR as the average number of gene-sets with equal or smaller *p* value over permutations. We have selected 25% as the empirical FDR significance threshold. Significant gene-sets were visualized using the Enrichment Map Cytoscape plugin (Merico *et al.* 2010).

## Results

### Detection of CNVs in ASD cases and controls

**Figure 1:**
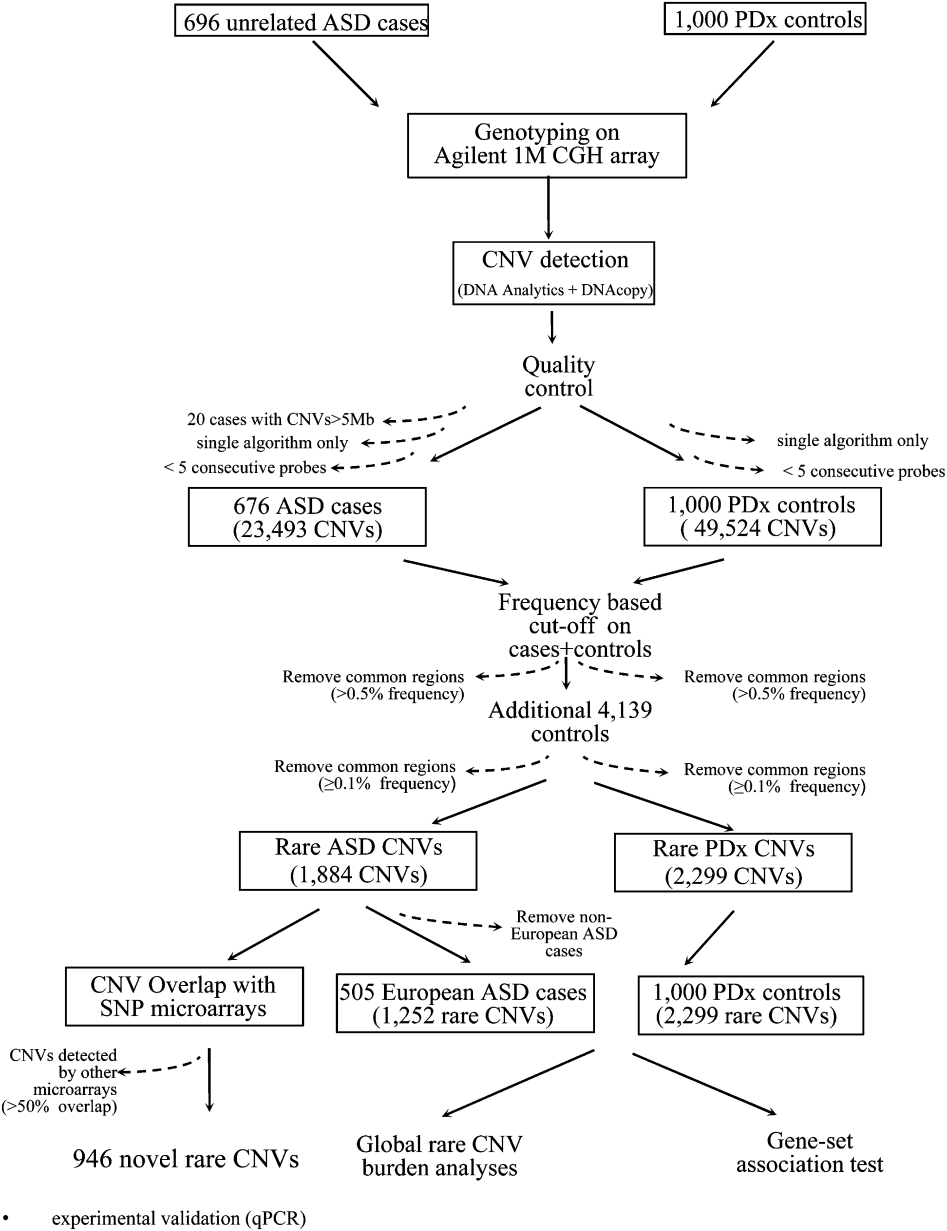
CNV analysis workflow. The ASD cases and controls were typed using the Agilent 1M CGH array, and CNVs were identified using two algorithms, DNA Analytics and DNAcopy. CNVs detected by both algorithms were defined as the stringent dataset and were used for novel rare variant discovery. Rare variants were defined as described in the *Materials and Methods*. The 1884 rare ASD CNVs (as reported in Table S2) were compared with the CNVs obtained from SNP microarray studies and this resulted in identification of 946 rare CNVs that were novel to the Agilent 1M CGH platform. The rare CNVs from only European ASD cases (505 cases) were then used for global rare CNV burden analysis and gene-set association tests by comparison to 1000 PDx controls.

Of the 696 unrelated ASD cases examined by array CGH, 20 were found to carry CNVs larger than 5 Mb (Supporting Information, Table S1) and excluded from further analysis. Four of these cases were known to have Down syndrome as well as an ASD diagnosis, 14 carried large chromosomal abnormalities previously detected through karyotyping and genotyping with SNP microarrays, and two cases harbored cell line artifacts. The rare stringent CNVs from the remaining 676 unrelated ASD cases were used for gene burden analysis, gene-set association tests and comparison with CNV data from other SNP microarrays to identify novel CNVs (Figure 1, Table 1).

**Table 1:** Summary statistics of stringent CNVs found in ASD and PDx control data sets

	Unrelated ASD Cases	PDx Controls
No. samples	676	1000
No. males/females	560/116	502/498
No. stringent CNVs*^a^*	23,493	49,524
Mean no. CNVs/ sample ± SD*^b^*	34.75 ± 6.40	49.52 ± 5.95
Median no. CNVs/sample	34	49
Mean CNV size, kb ± SD	97.75 ± 229.412	87.50 ± 198.59
Median CNV size, kb	30	30
% gain /% loss	42.36/57.64	48.24/51.76
No. CNVs of size >1 Mb (%)	471 (2)	942 (1.90)
No. CNVs of size from 100 Kb to 1 Mb (%)	3715 (15.81)	7982 (16.12)
No. CNVs of size from <100 Kb (%)	19,307 (82.18)	40,600 (81.98)

ASD, autism spectrum disorder; CNV, copy number variations; PDx, control cohort DNA samples.

a CNVs detected in the same individual using two algorithms, DNA Analytics and DNAcopy, were merged with the outside probes used as boundaries and defined as stringent CNV dataset containing at least five consecutive probes. Samples containing CNVs larger than 5 Mb were excluded (Table S1).

b We observed a significant difference in average number of CNVs per sample in PDx controls than in ASD cases (One-tailed exact Wilcoxon test *p* value < 2.2e-16) possibly due to reference bias.

We also examined the CNV content of the 1000 PDx controls and observed a significantly higher average number of CNVs per sample (*p* value < 2.2e-16) compared with the ASD cases (Table 1). This finding is likely attributable to the use of a different reference DNA, a single sex-matched individual, in the PDx CGH experiments rather than a pool of 50 sex-matched individuals used as a reference in the ASD CGH experiments. However, when we focused on rare variants (as defined in the materials and methods section), we found a smaller yet significant difference in the opposite direction (*p* value 0.002287; Table 2). The presence of a significant change in the relation between class (ASD, control) and CNV number after restricting to rare variants was further confirmed using a quasi-Poisson and linear regression model (class variable and rare variant filter variable interaction *p* value < 2.2e-16). Of the 676 unrelated ASD cases and 1000 PDx controls, 630 ASD cases (93%) and 896 PDx controls (90%) had at least one rare variant detected on the CGH 1M array (Table 2).

**Table 2:** Summary statistics of rare CNVs found in ASD and PDx control data sets

	Unrelated ASD Cases	PDx Controls
No. samples with ≥1 rare CNV	630	896
No. CNVs	1884	2299
Mean no. CNVs/sample ± SD*^a^*	2.99 ± 1.69	2.57 ± 1.44
Median no. CNVs/sample	3	2
Mean CNV size, kb ± SD	109.79 ± 314.56	95.62 ± 215.74
Median CNV size, kb	28.74	30.43
% gain/% loss	39.81/60.19	44.04/55.96
No. CNVs of size >1 Mb (%)	30 (1.59)	24 (1.04)
No. CNVs of size from 100 Kb to 1 Mb (%)	362 (19.21)	498 (21.66)
No. CNVs of size from <100 Kb (%)	1492 (79.19)	1777 (77.29)

ASD, autism spectrum disorder; CNV, copy number variations; PDx, control cohort DNA samples.

a We observed significant difference in average number of rare CNVs in ASD cases compared with PDx controls (one-tailed exact Wilcoxon test *p* value 0.002287).

### CNV comparison with other microarray platforms

**Figure 2:**
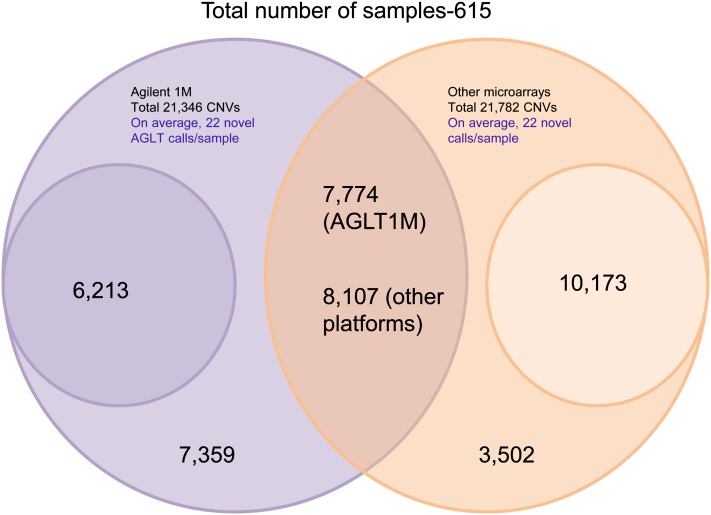
A Venn diagram showing comparison of Agilent 1M CNV calls with those detected by other SNP microarray platforms including Illumina 1M single/duo array, Affymetrix500K, Affymetrix6.0, and Illumina Omni 2.5M array for a total of 615 samples. Agilent 1M CGH data yielded 21,346 stringent CNVs and the SNP (other) microarray platforms yielded 21,782 stringent CNVs. A total of 7774 CNVs (36%) detected by the Agilent 1M array (AGLT1M) were detected by other platforms, whereas 8107 CNVs (37%) detected by other microarrays were detected by the Agilent 1M array. A total of 6213 (29%) Agilent 1M CNVs have less than 5 probe coverage in other SNP microarray platforms and 10,173 (47%) CNVs from the other SNP microarray platforms have less than 5 probe coverage in Agilent 1M array and are shown as smaller circles in this figure.

Of the 676 unrelated ASD cases, 615 were genotyped previously with SNP microarrays including 26 cases on Illumina Human Omni 2.5M-quad (2.5 million probes), 234 cases on Affymetrix Genome-Wide Human SNP Array 6.0 (1.8 million probes), 262 cases on Illumina Human 1M single infinium chip (1 million probes), 11 cases on Illumina 1M duo array (1 million probes), and 82 ASD cases were genotyped on lower resolution Affymetrix Mapping 500K chip set (500,000 probes). The other 61 unrelated ASD cases were only run on the Agilent 1M array and thus not included in this CNV platform comparison analysis. For the 615 samples run on both SNP and CGH array platforms, we performed a sample-level 50% one-way overlap of stringent Agilent 1M CNVs with the stringent CNVs from the SNP array platforms. We found that 64% of the Agilent 1M CNVs are novel with respect to the CNVs detected on the SNP microarrays (Figure 2). A more detailed comparison of CNVs detected by the 1M CGH array *vs.* the various SNP array platforms is shown in Table 3. For example, a comparison between CNVs detected using similar resolution arrays Agilent 1M and Illumina 1M showed that, on average, 24 novel CNVs/sample were detected by the Agilent 1M CGH array whereas only eight novel CNVs/sample were detected by the Illumina 1M SNP array. This platform comparison analysis suggests that use of multiple microarray platforms provides complementary data as every microarray platform detects a unique set of novel CNVs.

**Table 3:** Details of platform comparison

	Arrays	No. Samples	No. Stringent CNVs	Average CNVs/Sample	Average CNV Size, kb	No. Overlapping calls	% Overlap/ Validation*^a^*	% CNVs With Insufficient Probes in Other Array	Novel CNVs/Sample
1	Agilent 1M	82	2747	33.5 ± 6.11	107	183	6	67.5	31.27 ± 5.38
	Affy 500K	82	289	3.52 ± 2.28	520	189	65	6	2.17 ± 1.22
2	Agilent 1M	262	8971	34.24 ± 6.45	97	2725	30	38	23.84 ± 5.30
	Ilmn 1M single	262	4779	18.24 ± 4.69	110	2729	57	59.12	7.82 ± 3.11
3	Agilent 1M	11	413	37.55 ± 6.76	83	99	24	39.70	28.55 ± 5.13
	Ilmn 1M duo	11	169	14.36 ± 4.32	84	108	64	67.21	5.54 ± 1.81
4	Agilent 1M	234	8270	35.49 ± 6.23	98	4439	53	5.6	16.44 ± 4.27
	Affy 6.0	234	15,315	65.45 ± 8.87	80	4701	34	77.46	45.35 ± 7.39
5	Agilent 1M	26	945	36.35 ± 6.27	108	328	35	33	23.73 ± 4.63
	Ilmn Omni 2.5M	26	1230	47.31 ± 9.77	61	380	31	81.4	32.69 ± 8.57

ASD, autism spectrum disorder; CNV, copy number variations; PDx, control cohort DNA samples.

a % overlap/validation- refers to the percentage of CNVs that were also detected by the other array. For example, for the first platform comparison between Agilent 1M and Affy500K arrays, only 6% of the CNVs detected by Agilent 1M were also detected by Affy500K while 65% of the CNVs detected by Affy500K were also detected or validated by Agilent 1M array.

Rare ASD CNVs that were not detected by SNP microarrays but were detected by the Agilent 1M CGH array were defined as novel rare CNVs (946 of 1884 rare CNVs detected on the CGH 1M array). We examined the size distribution of these 946 novel rare CNVs detected in the ASD cohort and found that approximately 75% of them are <30 kb in size (Figure S1). These smaller CNVs that went undetected by SNP microarrays were missed due to insufficient probes coverage at these loci and because of the lower signal-to-noise often observed for SNP array platforms (*e.g.*, SNP-based arrays are optimized for robust genotyping call rates, which may minimize quantitative probe response to copy number variation). Only 25% of the novel, rare CNVs were >30 kb in size and were mostly missed by previous studies due to insufficient probe coverage, CNV-calling parameters and analysis algorithms chosen to define a CNV as stringent.

### Rare ASD-specific CNVs

**Figure 3:**
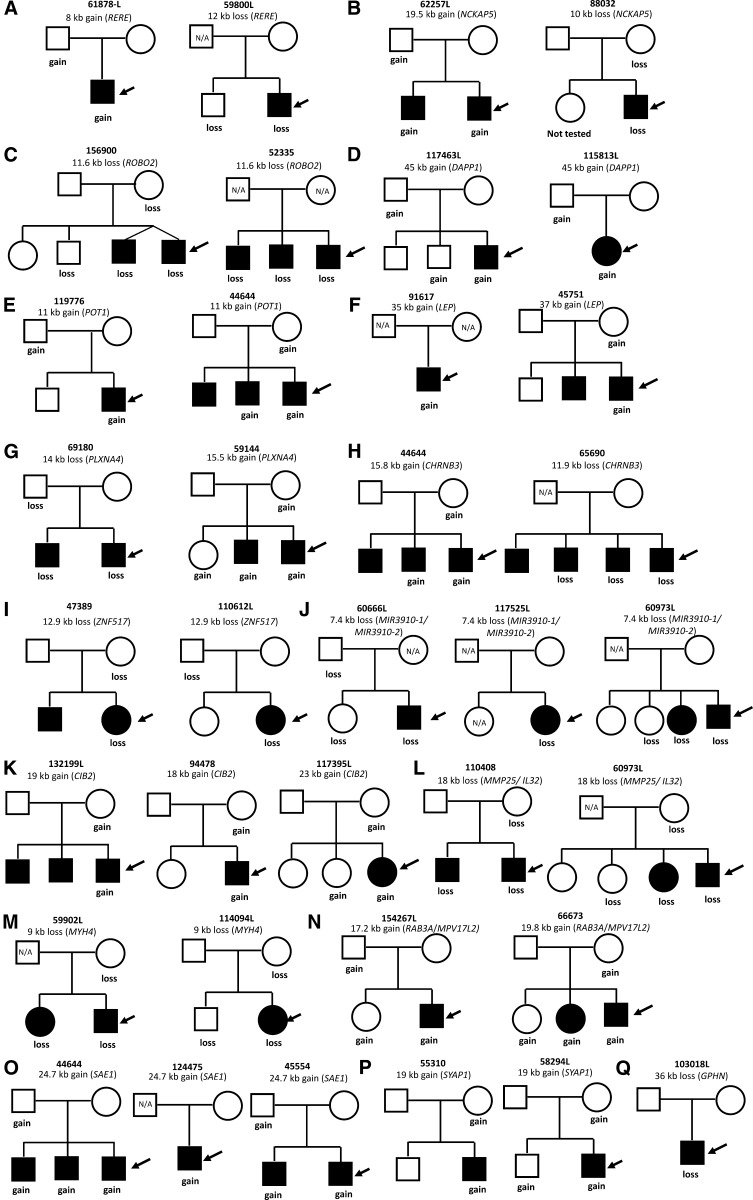
Pedigrees (A-Q) represent ASD families with overlapping/recurrent CNVs in novel loci and a *de novo* CNV event (from Table 4). The open symbols represent unaffected individuals, filled symbols represent individuals with ASD diagnosis and arrows indicate the probands. Individuals from which DNA was not available (N/A) for testing are denoted inside the symbols.

**Figure 4:**
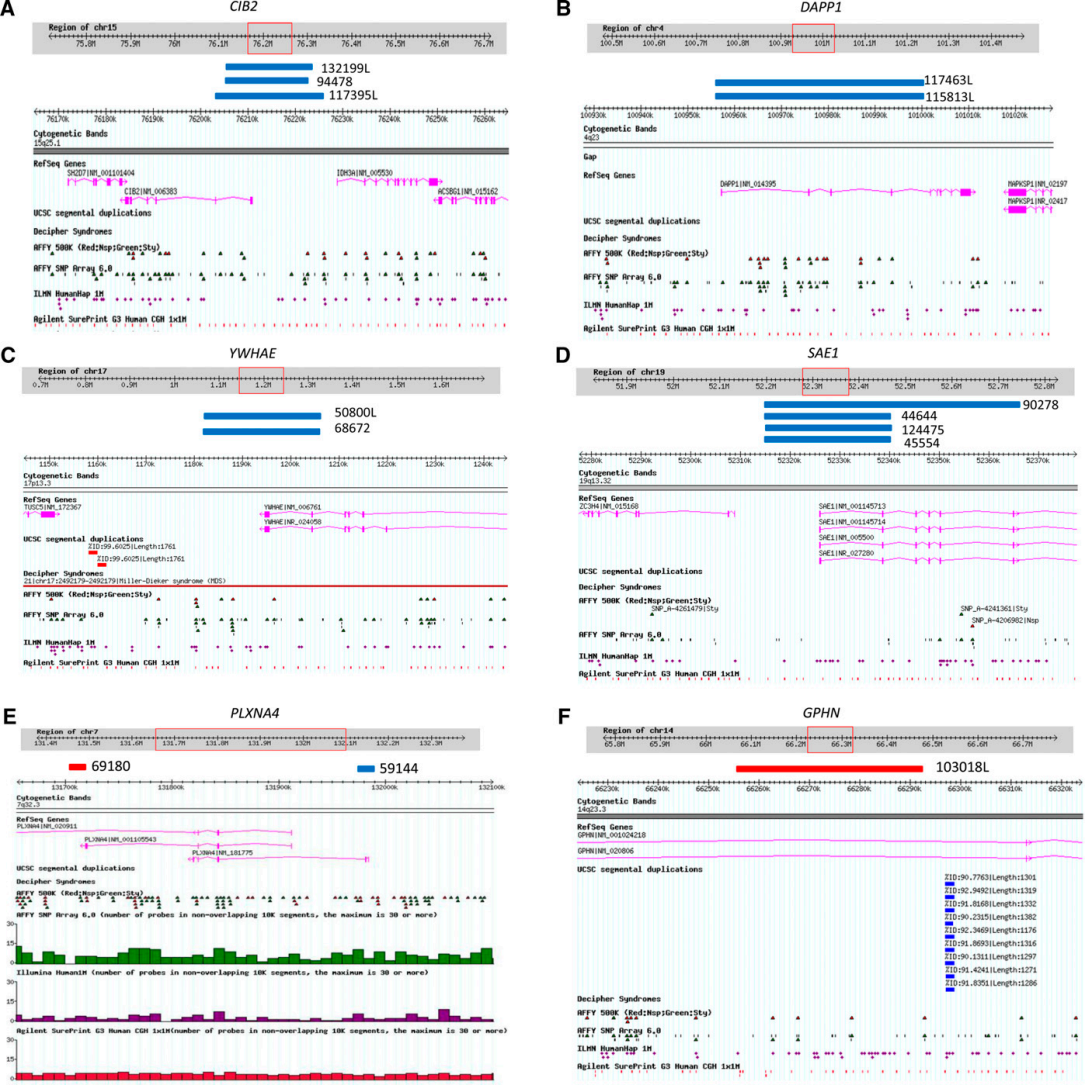
Genome browser views of a subset of novel rare CNVs occurring in two or more ASD cases or are *de novo* (from Table 4). The genome coordinates are from genome build 36 (hg18) and, in each panel, ASD case ID numbers are listed next to blue bars (denoting a duplication/gain) or red bar (denoting a deletion/loss). Each panel also shows, in separate tracks, the RefSeq genes, UCSC segmental duplications, and probe distributions for the different microarray platforms used for CNV detection (Affy500K, Affy6.0, Illumina 1M, and Agilent 1M). (A) Maternally inherited duplications at the 15q25.1 locus encompassing an exon of the *CIB2* gene in three unrelated ASD cases. (B) Paternally inherited duplications at the 4q23 locus encompassing several exons of the *DAPP1* gene in two unrelated ASD cases. (C) Maternally inherited duplications at the 17p13.3 locus encompassing exons of the *YWHAE* gene in two unrelated ASD cases. (D) Duplications at the 19q13.32 locus encompassing exons of *SAE1* gene in four unrelated ASD cases, three of which are novel and the fourth (ASD case 90278) was previously detected using an Affymetrix 6.0 array. (E) A paternally inherited deletion and a maternally inherited duplication at the 7q32.3 locus encompassing exons of the *PLXNA4* gene (F). A *de novo* deletion at the 14q23.3 locus encompassing an intronic region of the *GPHN* gene in one ASD case.

Rare ASD-specific CNVs were defined using a total of 5139 controls (1000 PDx controls summarized in Table 2 and 4139 in-house controls previously reported in Krawczak *et al.* 2006, Stewart *et al.* 2009, and Bierut *et al.* 2010). A total of 1884 rare CNVs were detected in ASD cases, of which 946 of them were novel as compared with previous SNP microarray studies (Table S2). Using qPCR, we validated 117 of 132 (88.6%) novel and rare stringent CNVs that were tested. Of the 946 novel rare ASD CNVs, 57 CNVs are reported in Table 4 that correspond to overlapping CNVs in two or more unrelated ASD cases (32 cases at 14 loci), recurrent CNVs (*i.e.*, same breakpoints) in two or more unrelated ASD cases (24 cases at 11 loci), or are a *de novo* event [1 case (Table 4)]. Some of the overlapping/recurrent CNVs impacted previously identified ASD genes such as *DPYD*, *RGS7*, *NRXN1*, *CNTNAP5*, *ERBB4*, *GRM8*, *NRXN3*, *YWHAE*, and *DMD* (Serajee *et al.* 2003; Wu *et al.* 2005; Autism Genome Project Consortium 2007; Marshall *et al.* 2008; Bruno *et al.* 2010; Pagnamenta *et al.* 2010; Pinto *et al.* 2010; Carter *et al.* 2011; Vaags *et al.* 2012), whereas others were novel, including *RERE*, *NCKAP5*, *ROBO2*, *DAPP1*, *POT1*, *LEP*, *PLXNA4*, *CHRNB3*, *ZNF517*, *MIR3910-1/MIR3910-2*, *CIB2*, *MMP25/IL32*, *MYH4*, *RAB3A/MPV17L2*, *SAE1*, and *SYAP1* (Figures 3 and 4).

**Table 4:** Novel recurrent/overlapping ASD CNVs (≥ 2 cases) and a *de novo* CNV

No.	Chromosome	Sample	Gender	Size, bp*^a^*	CNV	Origin	Genes*^b^*	Other Rare Variants*^c^*
1	1p36.23	61878-L	M	8602	Gain	Paternal	*RERE* intronic	7q31.1 (231.3 kb) loss not in any gene
	1p36.23	59800L	M	12,682	Loss	Unknown*^d^*	*RERE* intronic	2q31.3 (126.4 kb) loss not in any gene;11q14.3 (62.6 kb) loss not in any gene;13q12.13 (9.8 kb) Gain not in any gene;16p13.3 (34 kb) loss *LOC342346*
2	1p21.3	82302	M	11,699	Loss	Paternal	*DPYD* intronic	13q33.2 (25.2 kb) loss not in any gene;18p11.21 (1010.9 kb) gain *CXADRP3*, *ANKRD30B*, *MC2R*, *LOC284233*, *ZNF519*, *POTEC*, *MC5R*
	1p21.3	82062L	M	10,405	Loss	Maternal	*DPYD* exonic	7q31.1 (8.4 kb) Gain not in any gene
3	1q43	117370L	F	13,664	Loss	Unknown*^d^*	*RGS7* intronic	7q21.2 (505.2kb) loss *LRRD1*, *MTERF*, *AKAP9*, *CYP51A1*;21q21.2 (37.3 kb) gain not in any gene
	1q43	52401	M	13,664	Loss	Paternal	*RGS7* intronic	15q25.2 (42.8 kb) gain *LOC648809*;Xp22.33 (25.3kb) loss *DHRSX*
4	2p16.3	87396	F	6925	Loss	Maternal	*NRXN1* intronic	1q32.1 (18.9 kb) Gain not in any gene;2p16.3 (106.6kb) loss not in any gene;5q33.1 (349.1 kb) loss not in any gene
	2p16.3	78391	F	13,027	Loss	Maternal	*NRXN1* exonic	1q23.1 (21.7 kb) loss *OR6K2*;5p15.33 (56.2 kb) gain not in any gene;5q34 (35.3 kb) loss not in any gene;11p12 (16.5 kb) loss not in any gene;12q21.32 (13.6 kb) loss *MGAT4C*;18q12.1 (58.4 kb) loss not in any gene
	2p16.3	122686L	M	8739	Loss	Paternal	*NRXN1* intronic	1q44 (229 kb) gain *ZNF238*;3q29 (320.2 kb) loss *APOD*, *ACAP2*, *PPP1R2*;8q24.3 (189.8 kb) gain *ZNF7*, *RPL8*, *ZNF251*, *ZNF250*, *COMMD5*, *ZNF34*, *ZNF517*
	2p16.3	L384	M	81,779	Loss	Paternal	*NRXN1* exonic	1q25.3 (24 kb) loss *MR1,STX6*;12p12.1 (10.7 kb) loss not in any gene;13q22.1 (11.3 kb) loss *KLF12*;15q15.3 (14.2 kb) loss *CASC4*;17p13.1 (18.3 kb) loss *KDM6B*;19p13.12 (24.8 kb) Gain *CYP4F22*
5	2q14.3	111520L	M	17,780	Loss	Maternal	*CNTNAP5* intronic	3q23 (56.4 kb) loss *SPSB4*;8p12 (37.6 kb) loss not in any gene
	2q14.3	129914	M	10,249	Loss	Maternal	*CNTNAP5* intronic	1p36.22 (15 kb) Gain *CORT,APITD1-CORT,APITD1*;2p13.1 (8.5 kb) loss *C2orf65*;4q28.1 (51.7 kb) Gain not in any gene;Xq11.1 (18.7 kb) loss not in any gene
6	2q34	90188	F	6323	Loss	Maternal	*ERBB4* intronic	2q33.1 (60.5 kb) gain not in any gene;17p13.2 (174.7 kb) loss *TEKT1*, *ALOX12P2*, *XAF1 ,FBXO39*
	2q34	138145L	M	11,613	Loss	Not qPCR tested	*ERBB4* intronic	4q22.1 (97.3 kb) loss *MMRN1*; 13q31.3 (22.1 kb) loss *GPC5*
7	2q21.2	62257L	M	19,521	Gain	Paternal	*NCKAP5* exonic	11p14.3 (36k b) gain *LUZP2*
	2q21.2	88032	M	10,109	Loss	Maternal	*NCKAP5* intronic	2q22.1 (23.6 kb) loss *THSD7B*;3p26.3 (57.6 kb) loss not in any gene;7q32.3 (112 kb) Gain *CHCHD3*;8p12 (14.1 kb) loss not in any gene;Xq21.1 (108.8 kb) gain not in any gene
8	3p12.3	156900	M	11,610	Loss	Maternal	*ROBO2* intronic	15q15.1 (8.7 kb) loss *EHD4*
	3p12.3	52335	F	11,610	Loss	Unknown*^d^*	*ROBO2* intronic	8q21.13 (36.3 kb) loss not in any gene;14q23.3 (96 kb) loss not in any gene;17q25.3 (11.5 kb) loss not in any gene
9	4q23	115813L	F	45,723	Gain	Paternal	*DAPP1* exonic	17q12 (976.7 kb) gain *CCL2*, *ACCN1*, *CCL13*, *CCL7*, *CCL11*, *CCL8*, *C17orf102*, *CCL1 ,TMEM132E*
	4q23	117463L	M	45,723	Gain	Paternal	*DAPP1* exonic	1q42.13 (110.3 kb) gain *RNF187*, *HIST3H3*, *HIST3H2BB*, *HIST3H2A*;6p22.1 (234.7 kb) gain *SCAND3*, *GPX5*, *GPX6*
10	7q31.33	119776	M	11,094	Gain	Paternal	*POT1* exonic	7q34 (15.4 kb) loss not in any gene
	7q31.33	44644	M	11,094	Gain	Maternal	*POT1* exonic	8p11.21 (15.9 kb) gain *CHRNB3*;12p13.33 (35.2 kb) loss *CACNA2D4*;16q22.3 (9.7 kb) loss not in any gene;17p13.2 (12.4kb) loss *TM4SF5*;19q13.32 (24.7 kb) gain *SAE1*
11	7q31.33	128860	M	34,699	Gain	Paternal	*GRM8* exonic	7p22.1 (14.8 kb) loss not in any gene;18q22.1 (12.5kb) gain *CCDC102B*
	7q31.33	146436L	M	16,963	Gain	Maternal	*GRM8* intronic	9q34.3 (38.7 kb) loss *PNPLA7*
	7q31.33	130293	F	21,856	Loss	Maternal	*GRM8* intronic	13q21.1 (100 kb) loss not in any gene;Xq27.3 (33.2 kb) gain not in any gene
12	7q32.1	91617	M	35,669	Gain	Unknown*^d^*	*LEP* exonic	17q23.2 (9.8 kb) loss *BCAS3*
	7q32.1	45751	M	37,923	Gain	Maternal	*LEP* exonic	16q24.2 (12 kb) gain *JPH3*
13	7q32.3	69180	M	14,254	Loss	Paternal	*PLXNA4* exonic	1p36.32 (40.7 kb) gain not in any gene;13q12.11,13q12.12 (383.8 kb) gain *BASP1P1*;15q22.2 (21.9 kb) loss not in any gene
	7q32.3	59144	M	15,521	Gain	Maternal	*PLXNA4* UTRs	2q14.2 (8.5 kb) loss not in any gene;5q35.3 (22.2 kb) gain *COL23A1*
14	8p11.21	44644	M	15,873	Gain	Maternal	*CHRNB3* intronic	7q31.33 (11.1 kb) gain *POT1*;12p13.33 (35.2 kb) loss *CACNA2D4*;16q22.3 (9.7 kb) loss not in any gene;17p13.2 (12.4 kb) loss *TM4SF5*;19q13.32 (24.7kb) gain *SAE1*
	8p11.21	65690	M	11,965	Loss	Unknown*^d^*	*CHRNB3* intronic	4q32.1 (8.8 kb) loss not in any gene;7q11.23 (30.3 kb) Gain not in any gene;15q26.2 (13.8 kb) loss not in any gene;17q12 (25.7 kb) loss not in any gene
15	8q24.3	47389	F	12,937	Loss	Maternal	*ZNF517* exonic	8q11.23 (48 kb) loss *MRPL15*;13q22.1 (68.3kb) loss *PIBF1*
	8q24.3	110612L	F	12,937	Loss	Paternal	*ZNF517* exonic	12q13.11 (12.2 kb) loss COL2A1
16	9q22.31	60666L	M	7480	Loss	Paternal	*MIR3910-1*,*MIR3910-2*	6q16.1 (87.9 kb) loss *KLHL32*;10q23.31 (10.6 kb) loss *STAMBPL1*;12q21.1 (20.5 kb) loss not in any gene
	9q22.31	117525L	F	7480	Loss	Unknown^c^	*MIR3910-1*,*MIR3910-2*	3p26.1 (24.8 kb) loss not in any gene
	9q22.31	60973L	M	7480	Loss	Maternal	*MIR3910-1*,*MIR3910-2*	7p21.3 (42.5 kb) gain *THSD7A*;10p12.33 (23.4 kb) loss not in any gene;16p13.3 (18 kb) loss *MMP25,IL32*;20p13 (4.3 kb) loss *PANK2*
17	14q24.3	102350	M	14,061	Loss	Maternal	*NRXN3* intronic	1q42.13 (10.2 kb) loss not in any gene;5q14.3 (96.8 kb) gain not in any gene;6p21.2 (194.5 kb) gain *DNAH8,GLP1R*
	14q31.1	95458L	M	289,711	Loss	Paternal	*NRXN3* exonic	10q25.3 (135 kb) gain *PNLIPRP3*
18	15q25.1	117395L	F	23,541	Gain	Maternal	*CIB2* exonic	12q21.2 (31.2 kb) loss not in any gene
	15q25.1	94478	M	17,639	Gain	Paternal	*CIB2* exonic	19p12 (573.7 kb) loss *ZNF826P*, *ZNF737*, *ZNF682*, *ZNF486*, *ZNF90*, *MIR1270-2*, *MIR1270-1*
	15q25.1	132199L	M	18,728	Gain	Maternal	*CIB2* exonic	2p14 (14.9 kb) loss not in any gene;6q12 (411.3 kb) loss *EYS*;7q35 (12.4 kb) Gain not in any gene;8q23.2 (217.3 kb) loss not in any gene
19	16p13.3	110408	M	18,045	Loss	Maternal	*MMP25*,*IL32*	3q13.2 (21.4 kb) loss not in any gene;4p13 (12.6 kb) loss *GRXCR1*
	16p13.3	60973L	M	18,045	Loss	Unknown*^d^*	*MMP25*,*IL32*	7p21.3 (42.5 kb) Gain *THSD7A*;9q22.31 (7.5 kb) loss *MIR3910-1*,*MIR3910-2*;10p12.33 (23.4 kb) loss not in any gene;20p13 (4.3 kb) loss *PANK2*
20	17p13.3	68672	M	24,812	Gain	Maternal	*YWHAE* exonic	4q28.3 (79.3 kb) Gain *LOC641365*;6p21.2 (24.3 kb) Gain *ZFAND3*;21q21.1 (11.7 kb) loss *C21orf34*
	17p13.3	50800L	M	24,812	Gain	Maternal	*YWHAE* exonic	7q31.1-q31.31 (11 Mb) loss encompasses 32 genes including *FOXP2*
21	17p13.1	59902L	M	9003	Loss	Maternal	*MYH4* exonic	2p21 (25.6 kb) loss *EML4*
	17p13.1	114094L	F	9003	Loss	Maternal	*MYH4* exonic	−
22	19p13.11	154267L	M	17,268	Gain	Paternal	*RAB3A*,*MPV17L2*	1p36.23 (11 kb) loss *GPR157*;4q21.1 (11.4 kb) gain not in any gene;7q31.33 (481.3 kb) loss not in any gene;8q21.3 (70 kb) loss *LRRC69*;13q34 (118.3 kb) gain *F10*,*MCF2L*,*F7*;14q31.3,14q31.2 (299.5 kb) loss not in any gene;Xp22.33 (262.1 kb) gain *DHRSX*
	19p13.11	66673	F	19,829	Gain	Maternal	*RAB3A*,*MPV17L2*	1p36.22 (9.5 kb) loss *PLOD1*;5q11.2 (32.2 kb) gain not in any gene;5q21.3 (8.8 kb) loss *FBXL17*;7q11.22 (27 kb) loss not in any gene;7q31.1 (19.2 kb) loss not in any gene;8p22 (24.1 kb) loss not in any gene;14q31.3 (30.2 kb) gain not in any gene;16q22.3 (33.2 kb) gain not in any gene
23	19q13.32	44644	M	24,729	Gain	Paternal	*SAE1* exonic	7q31.33 (11.1 kb) gain *POT1*;8p11.21 (15.9 kb) gain *CHRNB3*;12p13.33 (35.2 kb) loss *CACNA2D4*;16q22.3 (9.7kb) loss not in any gene;17p13.2 (12.4 kb) loss *TM4SF5*
	19q13.32	124475	M	24,729	Gain	Unknown*^d^*	*SAE1* exonic	17p13.1 (428.4 kb) gain *MYH1,MYH8,MYH13,GAS7,MYH4*
	19q13.32	45554	M	24,729	Gain	Paternal	*SAE1* exonic	1q43,1q42.3 (96.9 kb) gain *EDARADD*;8q21.3 (82.1 kb) loss not in any gene;12q21.1 (20.5 kb) loss not in any gene;Xq27.3 (82.2 kb) gain not in any gene
24	Xp22.2	55310	M	19,171	Gain	Maternal	*SYAP1* exonic	3q13.33 (29.1 kb) gain *ARHGAP31*;7q21.12 (12.3 kb) loss not in any gene;10q23.1 (23.3 kb) loss *NRG3*
	Xp22.2	58294L	M	19,171	Gain	Maternal	*SYAP1* exonic	7p21.3 (57.3 kb) loss not in any gene;8p22 (89.9 kb) gain *MTUS1*;11q25 (41.3 kb) gain not in any gene;Xp11.3 (6.3 kb) loss not in any gene
25	Xp21.1	100570L	M	6261	Loss	Maternal	*DMD* intronic	−
	Xp21.1	91548L	M	6917	Loss	Maternal	*DMD* intronic	2q31.2 (15.8 kb) loss *PDE11A*;Xp22.11 (124.1 kb) loss *DDX53*
26	14q23.3	103018L	M	36,180	Loss	*de novo*	*GPHN* intronic	−

ASD, autism spectrum disorder; CNV, copy number variations; F, female; M, male; qPCR, quantitative polymerase chain reaction; UTR, untranslated region.

a The CNVs were validated by qPCR. However, accurate breakpoints have not been identified. The size of the CNVs shown is as detected by microarrays.

b A CNV encompassing coding regions of the gene are defined as exonic, whereas those encompassing introns were defined as intronic. One of the CNVs intersected untranslated region of the gene (UTRs).

c Other rare variants present in the individual.

d Inheritance is unknown if the parents DNA were unavailable.

One of the ASD-specific CNVs was a maternally inherited duplication at 15q25.1 in three unrelated ASD cases (117395L, 94478, 132199L; Figure 4A) disrupting the exon of *CIB2* (Calcium and integrin binding family member 2; 3/696 cases *vs.* 0/5139 controls; FET two-tailed *p* = 0.001691). The transcript and protein of *CIB2* gene is found to be present mainly in the hippocampus and cortex of the brain (Blazejczyk *et al.* 2009). The encoded protein of this gene is shown to be involved in Ca^2+^ signaling, which controls a variety of processes in many cell types. In neurons, Ca2^+^ signaling maintains synaptic transmission, neuronal development and plasticity (Blazejczyk *et al.* 2009).

In another two unrelated ASD cases (115813L, 117463L), we identified a recurrent CNV of size 45.7 kb (2/696 cases *vs.* 0/5,139 controls; FET two-tailed *p* = 0.01421). The duplication disrupts four exons of the *DAPP1* (dual adaptor of phosphotyrosine and 3-phosphoinositides) gene at the 4q23 (Figure 4B) and the gene is suggested to be involved in signal transduction processes.

Another interesting recurrent CNV of size 24.8 kb was detected in two unrelated ASD cases (68672, 50800L), a duplication at 17p13.3 disrupting two exons of *YWHAE* (tyrosine 3/tryptophan 5-monooxygenase gene) gene, which presumably act via haploinsufficiency (Figure 4C). It was maternally inherited in both ASD cases, and an equivalent event based on our definition of overlap was not present in controls (2/696 cases *vs.* 0/5139 controls; FET two-tailed *p* = 0.01421). *YWHAE* belongs to the 14-3-3 family of proteins, which mediate signal transduction, and is highly conserved in both plants and mammals. Only microduplications in *YWHAE* gene have been reported in ASD. It has been shown that the phenotype of patients with a 17p13.3 microduplication involving *YWHAE* gene show autistic manifestation, behavioral symptoms, speech and motor delay, subtle dysmorphic facial features, and subtle hand-foot malformations (Bruno *et al.* 2010). It is also noteworthy that there were larger CNVs found in two PDx controls to intersect *YWHAE*. The two ASD cases were both of Asian descent, and we also have found other cases and controls of Asian descent bearing *YWHAE*-CNVs (M. Gazzellone, unpublished data), suggesting that the role of this gene in ASD need to be further explored.

In three unrelated male ASD probands (44644, 124475, 45554), we observed a recurrent novel CNV, a 24.7-kb duplication encompassing two exons of the *SAE1* (SUMO1 activating enzyme subunit 1) gene at the 19q13.32 locus (Figure 4D). The same CNV also was found in one control (3/696 cases *vs.* 1/5139 controls; FET two-tailed *p* = 0.00616). Interestingly, another duplication of size 50.8 kb disrupting six exons of *SAE1* was observed in a fourth unrelated ASD case (90278) in the present study and was also detected by previous SNP microarray study (Lionel *et al.* 2011). The *SAE1* gene is involved in protein sumoylation process and is shown to interact with the *ARX* gene, which is involved in Autistic disorder (Sherr 2003; Rual *et al.* 2005; Ewing *et al.* 2007; Gareau and Lima 2010; Wilkinson *et al.* 2010; Szklarczyk *et al.* 2011).

In two unrelated cases (69180, 59144), we identified overlapping CNVs impacting the *PLXNA4* (plexin A4) gene at the 7q32.3 locus (Figure 4E). One CNV is a 14.2-kb loss encompassing an exon of the gene and based on our overlap criteria is not observed in 5,139 controls, while the second CNV is a 15.5-kb gain encompassing untranslated regions of the gene and is observed in 2 of 5139 controls. *PLXNA4* is involved in axon guidance as well as nervous system development (Suto *et al.* 2003; Miyashita *et al.* 2004).

In the present study, only one *de novo* CNV was found (all other qPCR-validated CNVs, 116 of 117, were inherited from either parent) a 36-kb loss encompassing the intron of the *GPHN* (Gephyrin) gene at the 14q23.3 locus. This *de novo* CNV (Figure 4F) was found in a male ASD proband (103018L) and was not picked up on the previous SNP array (Pinto *et al.* 2010), and it was not found in any of the 5139 controls. Gephyrin is suggested to play a central organizer role in assembling and stabilizing inhibitory postsynaptic membranes in human brain (Waldvogel *et al.* 2003). In our other unpublished data, we have also identified another deletion encompassing several exons of *GPHN* in an unrelated ASD case and a *de novo* deletion encompassing exons of the gene in a schizophrenia case suggesting that *GPHN* gene could be a novel susceptibility gene playing a more general role in neurodevelopmental disorders. We believe the lack of novel, rare *de novo* CNVs captured in the present study is simply due to our study design because nearly all the *de novo* CNVs in this ASD cohort are relatively larger in size and therefore were already detected using SNP microarrays (Marshall *et al.* 2008; Pinto *et al.* 2010, Lionel *et al.* 2011, A. C. Lionel, unpublished data, *i.e.*, not reported in the present study).

We also detected other novel, rare CNVs present in only one unrelated ASD case (Table 5) in previously identified genes associated with ASD such as *CTNND2*, *CDH18*, *PARK2*, *NXPH1*, *MTHFD1*, and *NF1* [Table 5 (Williams and Hersh 1998; Marui *et al.* 2004; Glessner *et al.* 2009; Pinto *et al.* 2010; Griswold *et al.* 2011; Salyakina *et al.* 2011)]. Novel, rare CNVs occurring in genes known to be associated with other neurodevelopmental disorders (*e.g.*, *KIRREL3*) or potentially playing a role in neurodevelopment also were found (*e.g.*, *CTNNA2*, *NDST1*, *SLC24A2*, *NFIB*, *APLP2*, *ATP2C2*, *CECR2*, *DAGLA*, and *UPB1*).

**Table 5:** Other singleton novel rare CNVs

Chromosome	Sample	Gender	Size, bp*^a^*	CNV	Origin	Genes*^b^*	Other Rare Variants*^c^*
1p36.11	88253	M	16,863	Loss	Maternal	*WASF2* intronic	9p21.3 (13.1 kb) loss *KIAA1797*;9q22.31 (10.7 kb) loss not in any gene
1p34.1	94073	F	9205	Loss	Paternal	*PRDX1* exonic	2q35 (6.8 kb) loss 40972;8p23.1 (74.5 kb) loss *PINX1*,*MIR1322*
2p21	115818L	M	9068	Loss	Paternal	*PPM1B* exonic	16q22.3 (90.8 kb) gain not in any gene
2p16.3	75420	F	10,491	Gain	Maternal	*STON1-GTF2A1L*,*STON1*	2p16.3 (10.5 kb) gain *STON1-GTF2A1L*,*STON1*;2q14.1 (118.8 kb) gain *DPP10*;17q12 (84.3 kb) gain *GPR179*, *MRPL45*, *LOC440434*
2p12	100678L	F	52,069	Gain	Paternal	*CTNNA2* exonic	1p31.1 (53.5 kb) loss not in any gene;1q42.2 (5 kb) loss *FAM89A*;2p23.1 (7.4 kb) loss *GALNT14*;4p15.31 (19 kb) loss *SLIT2*;9p21.3 (65 kb) loss not in any gene;16q24.1 (12.4 kb) loss *ATP2C2*
2q11.2	47173L	F	780,104	Gain	Unknown*^d^*	11 genes	19q13.43 (843.3 kb) loss *TRAPPC2P1*, *ZNF835*,*USP29*,*ZNF17*, *ZNF71*, *ZNF749*, *ZNF264*, *LOC147670*, *VN1R1*, *AURKC*, *PEG3-AS1*, *ZIM2*, *ZIM3*, *ZNF304*, *ZNF805*, *ZNF547*, *ZNF543*, *MIMT1*, *ZNF460*, *DUXA*, *ZNF548*, *PEG3*;20q13.12 (15.1 kb) gain *CD40*
3p22.2	58016	M	38,181	Gain	Paternal	*LRRFIP2* exonic	6p22.1 (9.5 kb) loss *HIST1H2AG,HIST1H2BJ*;19q13.31 (78.2 kb) gain *TEX101*;21q21.1 (70.9 kb) loss *C21orf34*
3p14.2	L656	M	332,421	Loss	Maternal	*FHIT* exonic	1q32.1 (37 kb) loss *MDM4*;2p14 (26 kb) loss *FBXO48,APLF*;6q12 (16.3 kb) loss *EYS*;8q22.2 (38.3 kb) loss *VPS13B*;17q25.3 (46.2 kb) loss *CYTH1*;17q25.3 (32.7 kb) loss *LOC100294362*, *ENDOV*, *RNF213*;Xp11.3 (32.8 kb) loss *CXorf36*
3q13.31	52026	M	10,004	Loss	Maternal	*ZBTB20* intronic	7p11.2 (81.7 kb) gain *LOC650226*, *DKFZp434L192*;7q36.2 (70.7 kb) loss DPP6;13q31.2,13q31.3 (74.9 kb) gain not in any gene
3q13.33	55310	M	29,147	Gain	Paternal	*ARHGAP31* exonic	7q21.12 (12.3 kb) loss not in any gene;10q23.1 (23.3 kb) loss *NRG3*;Xp22.2 (19.2 kb) gain *SYAP1*
3q25.1	50002	F	7969	Gain	Unknown*^d^*	*WWTR1* intronic	1p36.22 (6.4 kb) loss *UBE4B*; 5p15.1,5p15.2 (300.2 kb) gain not in any gene;7p21.1 (20.7 kb) loss not in any gene;9p24.1 (10.2 kb) loss *KDM4C*;14q23.2 (8.4 kb) gain *MTHFD1*
3q26.2	46685	M	10,633	Loss	Paternal	*PRKCI* exonic	4q13.1 (32.6 kb) gain not in any gene
3q29	76066	M	27,516	Loss	Unknown*^d^*	*KIAA0226* exonic	17q25.3 (13.4 kb) gain *NARF*;22q11.21 (2762.8 kb) gain encompassing 65 genes
4p15.31	100678L	F	19,015	Loss	Maternal	*SLIT2* intronic	1p31.1 (53.5 kb) loss not in any gene;1q42.2 (5 kb) loss *FAM89A*;2p23.1 (7.4 kb) loss *GALNT14*;2p12 (52.1 kb) gain *CTNNA2*;9p21.3 (65 kb) loss not in any gene;16q24.1 (12.4 kb) loss *ATP2C2*
4q21.1	62345L	M	8527	Loss	Paternal	*SHROOM3* intronic	5q21.1 (20.8 kb) loss not in any gene;14q21.2 (59.5 kb) loss not in any gene
4q32.1	55360	M	16,194	Gain	Maternal	*FGA* exonic	9p21.3 (18 kb) gain *IFNA22P*;12p12.1 (18.9 kb) loss not in any gene;15q13.3 (36.3 kb) loss *MIR211*,*TRPM1*
5p15.2	51119	M	12,982	Loss	Unknown*^d^*	*CTNND2* intronic	—
5p14.3	167532	F	16,492	Gain	Maternal	*CDH18* intronic	11p15.4 (98.4 kb) loss *LOC283299*, *OR5E1P*, *OR5P3*, *OR5P2*;12q24.12 (12.7 kb) loss *ATXN2*
5q14.3	55262-L	M	665,114	Gain	Maternal	*LYSMD3*,*POLR3G*,*CETN3*,*MBLAC2*, *GPR98*	2q37.1 (119.8 kb) gain *NMUR1*,*C2orf57*;4q21.21 (22.7 kb) gain *ANTXR2*;14q22.2 (26.4 kb) gain *CDKN3*;22q13.31 (54.2 kb) loss not in any gene
5q22.2	59269L	F	25,834	Loss	Maternal	*MCC* exonic	22q12.1 (225.1 kb) loss *TTC28*;Xq11.1 (308.9 kb) gain *MTMR8*
5q31.3	66559	M	19,794	Gain	Unknown*^d^*	*TAF7*,*SLC25A2*	6q27 (307.6 kb) gain *C6orf118*, *PDE10A*;11q12.1 (24.3 kb) loss *OR5B2*, *OR5B12*;16q21 (81.9 kb) gain not in any gene
5q33.1	60560L	M	54,785	Gain	Paternal	*NDST1* exonic	20p13 (22.4 kb) gain *SNPH*;22q12.3 (77.4 kb) gain *SLC5A4*
6p22.1	58016	M	9549	Loss	Paternal	*HIST1H2AG*,*HIST1H2BJ*	3p22.2 (38.2 kb) gain *LRRFIP2*;19q13.31 (78.2 kb) gain *TEX101*;21q21.1 (70.9 kb) loss *C21orf34*
6q26	75744	M	17,712	Loss	Paternal	*PARK2* intronic	21q21.3 (8.1 kb) loss *NCRNA00189*
7p21.3	68687	M	258,183	Loss	Maternal	*NXPH1* exonic	1q32.1 (145.7 kb) gain *CAMSAP1L1*, *C1orf106*, *GPR25*;13q33.2 (63.9 kb) loss not in any gene;13q33.2 (69.6 kb) gain not in any gene
8p21.2	88810	M	28,281	Gain	Paternal	*ADAM7* exonic	6p22.1 (17.3 kb) gain not in any gene;19q13.43 (24.1 kb) gain *SLC27A5*,*ZBTB45*
9p22.3	131240	M	8341	Loss	Paternal	*NFIB* intronic	5p13.2 (51.9 kb) gain *PRLR*,*AGXT2*;6q25.1 (20.8 kb) loss *RAET1L*;8p12 (66.6 kb) loss *NRG1*;16p13.3 (16.5 kb) loss *HBM*, *HBA1*, *HBA2*, *HBQ1*
9p22.1	61180-L	M	7974	Loss	Paternal	*SLC24A2* exonic	3p21.31 (20.4 kb) loss *MIR425*, *DALRD3*, *MIR191*, *QRICH1*, *IMPDH2*, *NDUFAF3*;6q22.31 (296.4 kb) gain *C6orf204*, *PLN*, *BRD7P3*
9p13.3	59641L	F	190,658	Gain	Maternal	*PTENP1*,*PRSS3*,*UBE2R2*	6q24.1 (13.2 kb) loss *TXLNB*;17q24.1 (5.9 kb) loss *CEP112*;Xp21.1 (15.4 kb) gain *DMD*
10q25.1	115816L	M	9442	Loss	Maternal	*SORCS3* intronic	11q23.3 (9.2 kb) loss *CBL*;15q21.3 (58.8 kb) loss not in any gene
11q12.2	72816L	M	15,159	Loss	Paternal	*DAGLA* exonic	7q31.31 (19.4 kb) gain not in any gene;8q22.1 (19.8 kb) loss not in any gene;12q12 (25 kb) gain *KIF21A*;14q24.2 (8.5 kb) gain *RGS6*;22q12.1 (25.6 kb) loss *MIR1302-1*,*MYO18B*
11q23.3	96280L	M	19,424	Gain	Paternal	*VPS11* exonic	3p12.3 (123 kb) loss not in any gene;13q33.1 (74.2 kb) loss *FGF14*;15q11.2 (25.7 kb) loss not in any gene;Xp11.4 (39 kb) gain *GPR82*,*GPR34*,*CASK*
11q24.2	138952L	F	11,398	Loss	Maternal	*KIRREL3* intronic	4p13 (71.3 kb) gain not in any gene;11q14.1 (28.8 kb) gain not in any gene;14q31.2 (27.9 kb) loss not in any gene
11q24.3	98320L	M	8428	Loss	Maternal	*APLP2* exonic	1q31.1 (321.4 kb) loss not in any gene;4q13.1 (1326.4 kb) gain not in any gene;5q23.1 (42 kb) loss not in any gene;7q31.1 (98.6 kb) loss *IMMP2L*
12q15	59794L	M	9279	Loss	Maternal	*RAP1B* intronic	5q35.2 (8 kb) loss *ATP6V0E1*;14q32.2 (8.9 kb) loss *WDR25*;20p11.22 (148.7 kb) gain not in any gene
12q24.33	50280	M	202,302	Gain	Maternal	*LOC100130238*, *GALNT9*	3q21.3 (6.1 kb) loss *CCDC48*;12q24.33 (77.8 kb) gain *SFSWAP*
14q23.2	50002	F	8427	Gain	Unknown*^d^*	*MTHFD1* exonic	1p36.22 (6.4 kb) loss *UBE4B*;3q25.1 (8 kb) gain *WWTR1*;5p15.1,5p15.2 (300.2 kb) gain not in any gene;7p21.1 (20.7 kb) loss not in any gene;9p24.1 (10.2 kb) loss *KDM4C*
15q24.2	62261L	M	12,936	Loss	Paternal	*PTPN9* intronic	5q14.1 (15.3 kb) gain *BHMT2*;8q24.21 (45.3 kb) gain *GSDMC*
15q25.1	68388	F	7142	Loss	Paternal	*IL16* exonic	1p32.2 (567.4 kb) gain *DAB1*;10q22.3 (12.9 kb) gain *C10orf11*;22q12.1 (8.4 kb) loss not in any gene
16p13.3	47378	M	13,736	Loss	Maternal	*TRAP1* intronic	2p23.1 (42.7 kb) loss *CAPN14*,*EHD3*;3p25.1 (16.1 kb) loss *CAND2*;21q21.2,21q21.1 (75 kb) loss not in any gene
16p11.2	100564	F	546,709	Gain	Maternal	27 genes	8q12.3 (17.9 kb) loss *ASPH*;10q21.3 (47.3 kb) loss not in any gene;17p11.2 (186.2 kb) loss *LOC162632*, *CCDC144A*, *FAM106CP*, *KRT16P2*;Xq21.1 (130.3 kb) gain not in any gene
16q24.1	100678L	F	12,352	Loss	Maternal	*ATP2C2* exonic	1p31.1 (53.5 kb) loss not in any gene;1q42.2 (5 kb) loss *FAM89A*;2p23.1 (7.4 kb) loss *GALNT14*;2p12 (52.1 kb) gain *CTNNA2*;4p15.31 (19 kb) loss *SLIT2*;9p21.3 (65 kb) loss not in any gene
16q24.2	68711	M	17,969	Loss	Maternal	*KLHDC4* exonic	2p23.1 (61.4 kb) loss *LCLAT1*;4p15.1 (48 kb) loss not in any gene;6q21 (14.6 kb) gain not in any gene;7p21.3 (87.4 kb) loss *VWDE*;12p13.2 (8 kb) gain *KLRK1*, *KLRC4-KLRK1*
17p13.1	47387	M	7758	Gain	Maternal	*NDEL1*, *MYH10*	2p23.1 (7.4 kb) loss *GALNT14*;2q22.1 (299.4 kb) loss *THSD7B*;5q31.3 (29.9 kb) loss *PCDHB9*, *PCDHB8*, *PCDHB7*, *PCDHB16*
17q11.2	87042	F	14,177	Loss	Maternal	*NF1* exonic	−
17q25.3	103818L	M	48,984	Gain	Maternal	*TIMP2* exonic	8q24.13 (54.1 kb) loss not in any gene
19q13.32	85287L	M	17,455	Loss	Maternal	*BBC3*,*MIR3190*, *MIR3191*	16q22.1 (36.5 kb) gain *DDX19A*, *DDX19B*;16q23.1 (16.2 kb) loss *WWOX*
19q13.33	168753	M	61,936	Loss	Maternal	*SHANK1*,*CLEC11A*	4p15.33 (5.4 kb) loss not in any gene
20p13	92540L	M	15,645	Loss	Paternal	*SLC23A2* exonic	2q34 (23 kb) loss *SPAG16*
22q11.21	118909L	F	7277	Loss	Unknown*^d^*	*CECR2* exonic	7q22.2 (12.4 kb) gain not in any gene;10q21.3 (8.1 kb) loss *CTNNA3*
22q11.22-q11.23	MM0177-3	M	656,280	Gain	Maternal	*RAB36*,*FBXW4P1*,*RTDR1*,*GNAZ*,*MIR650*,*IGLL5*,*BCR*	−
22q11.23	154266L	M	6784	Loss	Maternal	*UPB1*	11p14.3 (138.6 kb) loss not in any gene
Xq28	100676L	M	123,871	Gain	Maternal	*ZNF185*,*CETN2*, *NSDHL*	3q22.3 (11.5 kb) loss not in any gene;5p14.2 (12.2 kb) loss not in any gene;22q12.3 (35.6 kb) gain not in any gene

ASD, autism spectrum disorder; CNV, copy number variations; F, female; M, male; qPCR, quantitative polymerase chain reaction; UTR, untranslated region.

a These CNVs were validated by qPCR. However accurate breakpoints have not been identified. The size of the CNVs shown is as detected by microarrays.

b A CNV encompassing coding regions of the gene are defined as exonic, whereas those encompassing introns were defined as intronic.

c Other rare variants present in the individual.

d Inheritance is unknown if the parents DNA were unavailable.

**Figure 5:**
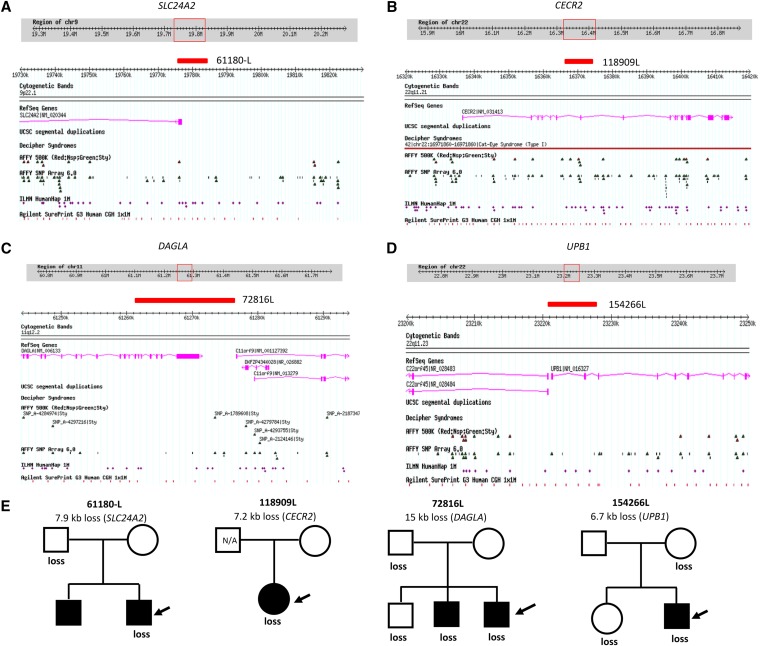
Genome browser view of a subset of the novel rare CNVs found in one ASD case that impact one or more exons (Table 5). The genome coordinates are from genome build 36 (hg18) and, in each panel, the ASD case ID number is listed next to a red bar (denoting a deletion/loss). Each panel also shows, in separate tracks, the RefSeq genes and probe distributions for the different microarray platforms used for CNV detection (Affy500K, Affy6.0, Illumina 1M and Agilent 1M). (A) Paternally inherited deletion at the 9p22.1 locus impacting the 5′-end of the *SLC24A2* gene. (B) Deletion at the 22q11.21 locus resulting in loss of an exon of the *CECR2* gene. (C) Paternally inherited deletion at the 11q12.2 impacting the 3′-end of the *DAGLA* gene. (D) Maternally inherited deletion at the 22q11.23 impacting exons near the 5′-end of the *UPB1* gene. (E) Pedigrees of ASD families for the CNVs in novel loci occurring in one ASD case that correspond to panels A-D. The open symbols represent unaffected individuals, filled symbols represent individuals with ASD diagnosis and arrows indicate the probands. Individuals from which DNA was not available (N/A) for testing are denoted inside the symbols.

A paternally inherited 7.9-kb deletion disrupting an exon of the *SLC24A2* [solute carrier family 24 (sodium/potassium/calcium exchanger), member 2] gene was observed at 9p22.1 in a male ASD case (61180-L; Figure 5A) but in none of the controls. The *SLC24A2* gene may play a role in neuronal plasticity (Li *et al.* 2006).

In a female ASD case (118909L), we observed a 7.2-kb loss disrupting an exon of the *CECR2* (cat eye syndrome chromosome region, candidate 2) gene at the 22q11.21 locus (Figure 5B), which was not observed in controls. We also detected a CNV in the same gene in ASD case 124632L reported in another study (Pinto *et al.* 2010). *CECR2* is a chromatin remodeling factor that has been proposed to play a role in embryonic nervous system development (Banting *et al.* 2005).

In another unrelated male ASD proband (72816L), we have identified a 15-kb paternally inherited deletion (Figure 5C) disrupting seven exons of the *DAGLA* (diacylglycerol lipase, alpha) gene at the 11q12.2 locus that is not found in controls. *DAGLA* is known to synthesize an endocannabinoid that has been associated with retrograde synaptic signaling and plasticity (Gao *et al.* 2010).

Another interesting gene is *UPB1* (ureidopropionase, beta) located at the 22q11.23 locus, in which a 6.7-kb deletion disrupting two exons has been found in a male ASD proband (154266L; Figure 5D). The deletion was not observed in any of 5139 controls. This gene is involved in the last step of the pyrimidine degradation pathway and deficiencies in *UPB1* have been associated with developmental delay (van Kuilenburg *et al.* 2004).

Finally, our study includes analysis of two ASD cases not previously run on SNP microarrays. In both cases, large-sized CNVs previously associated with ASD were found, a 546-kb maternally inherited 16p11.2 duplication (ASD case 100564) and a 656-kb maternally inherited 22q11.22-q11.23 duplication (ASD case MM0177-3) that overlaps with the 22q distal deletion region (Figure S2).

### Selection of ASD cases of European ancestry

To perform more robust downstream analyses (global rare CNV burden and gene-set association) on the ASD CNV findings in the present study, we used the SNP genotype data available on the 615 ASD cases to identify those of European ancestry based on MDS. We visualized the HapMap and ASD samples in a two-dimensional space using the first and second MDS components. The plot displayed three major clusters (Europeans, Eastern Asians, and Africans), with Mexicans and Indians distributed between the European and Eastern Asian cluster (Figure S3A). According to MDS components 1 and 2, ASD samples mostly clustered with Europeans, although some of them clearly clustered with other ethnicities. We defined a set of boundaries on the first and second MDS component, which identified all European samples, and only two Mexican samples (Figure S3B). Any ASD sample falling within these boundaries was considered to be European (505 of 615 total genotyped unrelated samples). We did not use the third MDS component, which is better at resolving ethnic subgroups but does not account faithfully for differences among major groups (*e.g.*, Europeans and Eastern Asians were very close to each other; Figure S3C). The rare variants from 505 European ASD cases along with 1000 European PDx controls were used further for rare CNV burden and gene-set association analyses to avoid any confounding issues due to different ancestries of samples.

### Global rare CNV burden analyses

To investigate global differences in CNV burden, we assessed the distribution of three CNV statistics in ancestry-matched European ASD case subjects compared with control subjects: (1) subject CNV number, (2) subject total CNV length, and (3) subject total number of CNV genes.

We observed significant differences in CNV number and total gene number for deletions but not for duplications (significance threshold: Wilcoxon test *p* value < 0.01; Table 6). In terms of magnitude, the total number of deletion genes was the largest difference found between the ASD and control subjects (ratio of means: 1.77). Considering that subjects were matched by platform type and other essential parameters, and also considering that previous authors found a stronger difference in deletion burden rather than duplication, the differences observed very likely translate to real biological differences. In addition, the relative difference in total gene number was larger than the relative difference in CNV number, an effect that is harder to explain by technical or experimental factors; because of the relatively large sample size, it is important to consider both the significance and magnitude of burden differences.

**Table 6:** Global burden of rare CNVs in European ASD cases *vs.* PDx controls

Type	Measure	Mean ASD	Mean Controls	Mean ratio ASD/controls	*P*-value
All	No. CNV	2.680942	2.565848	1.05	0.0575976
All	Total CNV size, kb	124.50	112.92	1.10	0.1804119
All	Total no. genes	3.850107	2.560268	1.50	0.2345081
Deletions only	No. CNV	1.958225	1.773793	1.10	0.0046012*^a^*
Deletions only	Total CNV size, kb	54.29	48.81	1.11	0.1043462
Deletions only	Total number of genes	1.741514	0.9834483	1.77	0.0087554*^a^*
Duplications only	No. CNV	1.549383	1.601266	0.97	0.7716051
Duplications only	Total CNV size, kb	101.66	96.02	1.11	0.3778319
Duplications only	Total no. genes	3.490741	2.495253	1.40	0.8564186

a Significant differences (*P* ≤ 0.01) are indicated.

We also assessed whether rare CNVs in genes that are causally implicated in ASD were enriched in cases over controls. The ASD gene list used comprised 110 genes compiled from the peer-reviewed literature (Betancur 2011). We observed significant enrichment of deletions impacting genes implicated in ASD [*p* = 8.896e-05, odds ratio = 20.59 with 95% confidence interval = 2.95-888.96] in ASD cases than controls (Table 7). There were 11 ASD cases with deletions in ASD candidate genes (Table S3) and all were experimentally validated by qPCR except three false-positive CNVs overlapping the *SYNGAP1* gene. The one *SYNGAP1* CNV that did validate is a *de novo* 112 kb deletion, which was described previously in the Pinto *et al.* 2010 study that disrupts *SYNGAP1* and encompasses four other genes. After removing the three false-positive CNVs in the *SYNGAP1* gene and testing for enrichment again, we still observed significant enrichment of deletions in genes implicated in ASD (*p* = 0.001585, odds ratio = 14.74 with 95% CI = 1.95−656.52) in ASD cases as compared with controls. The other ASD cases where we observed rare exonic loss in ASD candidate genes were *PTCHD1* (Marshall *et al.* 2008; Pinto *et al.* 2010), *VPS13B* (Pinto *et al.* 2010), *DMD* (Pinto *et al.* 2010), *DPYD* (novel to this study and as described in Carter *et al.* 2011), *SHANK2* (Pinto *et al.* 2010), *NF1* (novel to this study) and *NRXN1* (ILMN Omni 2.5 M array; A. C. Lionel, unpublished data).

**Table 7:** CNV burden in known ASD genes in cases *vs.* PDx controls

Type	Size*^a^*	Case counts	Control counts	ASD %	CT %	*p* value
All	110	14	15	4.307692	2.340094	0.0700680
Deletions	110	11	1	5.641026	0.288184	8.90E-05*^b^*
Duplications	110	3	14	1.369863	3.030303	0.9480197

ASD, autism spectrum disorder; CNV, copy number variations; PDx, control cohort DNA samples.

a Number of genes causally implicated in ASD (Betancur 2011).

b Significant differences (*p* ≤ 0.01) are indicated; ASD and CT (controls) % are based on the total number of subjects with at least one rare exonic CNV of the respective type.

### Gene-set association tests

**Figure 6:**
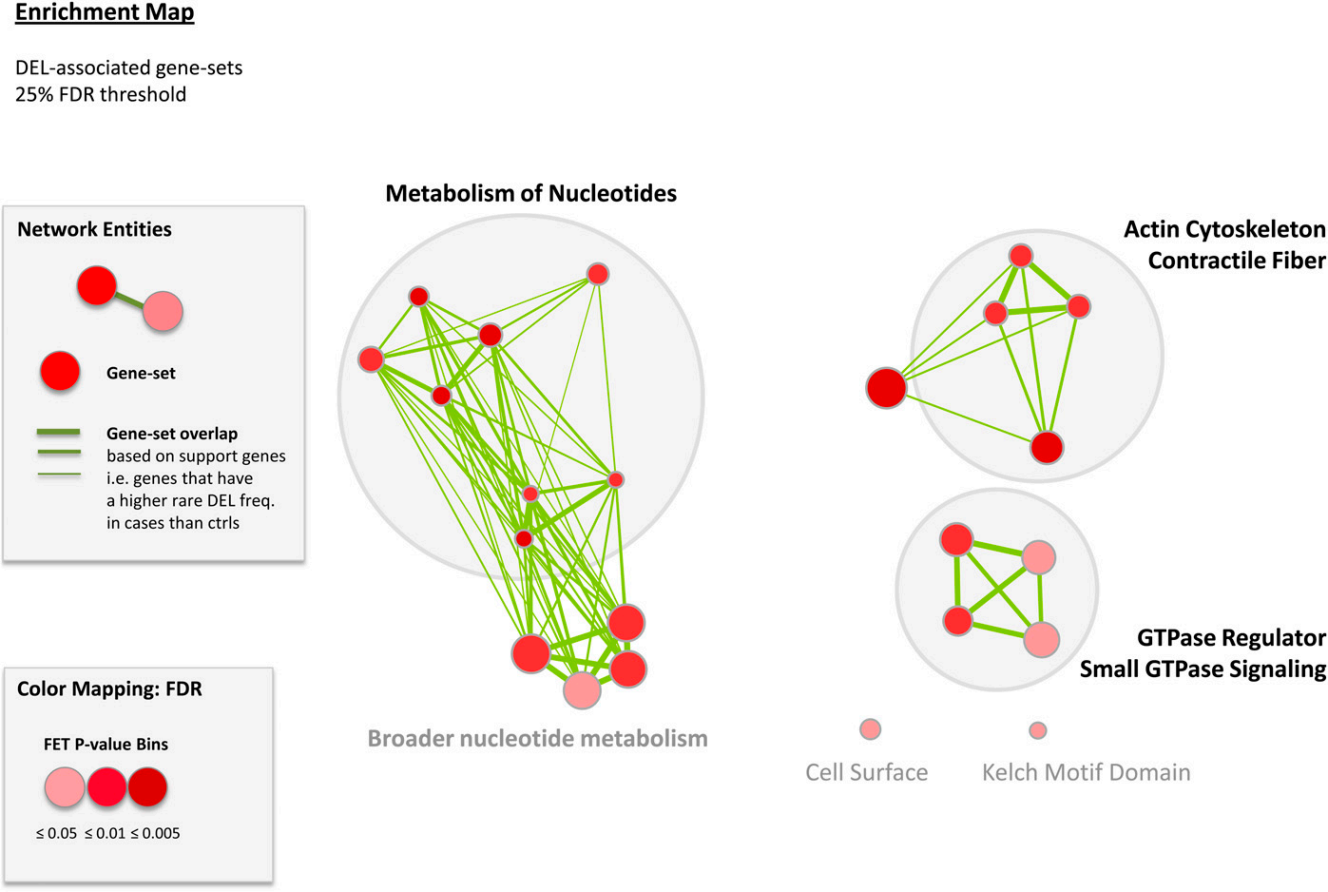
A gene-set functional enrichment map of ASD. Each node represents gene-sets with its size proportional to the total number of genes in each set. The map shows gene sets enriched for deletions with enrichment significance (permutation-based FDR) represented as a node color gradient.

We tested functional gene-sets for enrichment in ASD cases over controls to identify biological processes potentially involved in ASD. We found significant results only for deletions, which is in agreement with global burden results (Table 6). There were 23 gene-sets that had a permutation-based FDR < 25% in deletions and these were used to construct a functional map of ASD (Figure 6, Table S4). We identified several gene-set clusters, some of which were associated with ASD by earlier studies. For example, gene-sets involved in GTPase/Ras signaling pathways were previously reported in ASD (Meechan *et al.* 2009; Pinto *et al.* 2010). The novel pathway that has been discovered from this study is the gene-set enriched for nucleotide metabolism. The list of genes in the nucleotide metabolism pathway is shown in Table S5. To strengthen the conclusion that ASD variants impact genes involved in the nucleotide metabolism pathway, we performed experimental qPCR validation on this set of CNVs and found all were valid except one deletion arising due to cell line artifact in the *FHIT* gene. The confirmation of 14 of 15 genes (Table S5) based on qPCR validation of the microarray findings suggests that this pathway is not the result of false positives in the dataset.

## Discussion

Our high-resolution CGH data have identified multiple novel, rare CNVs in ASD cases that went undetected by SNP microarrays run on the same individuals; thus providing an additional valuable resource for ASD risk gene discovery and validation. Although hundreds of ASD susceptibility genes/loci (Betancur 2011) are now known, no single gene or locus accounts for more than 0.8% frequency of cases in a given cohort, with most contributing to less than 0.1% of ASD cases (Devlin and Scherer 2012). Moreover, other CNV studies (Pinto *et al.* 2010; Sanders *et al.* 2011) and recent exome sequencing studies (Neale *et al.* 2012; O’Roak *et al.* 2012; Sanders *et al.* 2012) suggest that perhaps several hundred autism risk genes may exist. These data indicate that to delineate all ASD risk genes and alleles, different experimental strategies will likely be required when assessing even larger sets of ASD patient collections.

We find that the Agilent CGH array is sensitive for detection of many smaller (<30 kb) CNVs, which are often missed by SNP microarrays even when probe coverage is sufficient at these loci. The results of this study suggest that currently available microarray platforms are complementary (*i.e.*, not all CNVs are captured by one platform/array design) and that the number and type of CNVs detected varies depending on microarray probe distribution, sample labeling and hybridization chemistries, and CNV detection algorithms used. The Agilent 1M array is designed exclusively for CNV detection and utilizes a more uniform probe distribution across the genome as compared with SNP arrays such as the Illumina 1M and Affymetrix 6.0 arrays. The median probe spacing of the Agilent 1M CGH array is 2.1 kb and a small proportion of probes correspond to regions that are non-unique in nature; that is, they map to multiple locations in the genome, with most targeted to segmentally duplicated regions. Therefore, the Agilent 1M CGH array is better at detecting CNVs in segmental duplicated regions compared to SNP arrays. Also, a better signal-to-noise ratio has been observed in CGH arrays compared to SNP arrays and is attributed to the use of longer probes [60-mers (Pinto *et al.* 2011)]. The SNP arrays are also biased toward known CNVs. However, in addition to copy number analysis, one of the advantages of using SNP arrays is the ability to genotype SNPs which is not possible using the CGH array from this study. Until a single technology (*e.g.*, whole-genome sequencing) is sufficiently robust to capture most genetic variants, including structural variants such as CNVs, use of multiple platforms will be advantageous. In some instances, the identification of previously undetected CNV variants will reveal pathogenic events relevant for clinical diagnosis of ASD (Miller *et al.* 2010; Scherer and Dawson 2011).

The discovery of additional rare variants in this present study led to the identification of a novel disease pathway of triphosphate nucleotide metabolism; specifically, purine and pyrimidine metabolism as a potential biological process involved in ASD. Several genes found to harbor a rare exonic loss in cases but not in controls have been previously associated to a neuropsychiatric or neurodevelopmental phenotype in human [*DPYD*, *UPB1* (van Kuilenburg *et al.* 2004, 2009)] and mouse [*UPP1*, *TYMP* (Haraguchi *et al.* 2002; López *et al.* 2009)] studies. Another interesting functional category revealed by this study is small GTPase signaling pathways, with genes such as *ARHGAP15*, *CDH13*, *NF1*, *RALGDS*, *SYNGAP1*, and *VAV3* harboring rare losses in cases but not in controls (Table S2). Rare mutations in the *ARHGAP15* gene have been reported to be associated with ASD (O’Roak *et al.* 2011); *NF1* and *SYNGAP1* genes are also implicated in ASD (Williams and Hersh 1998; Marui *et al.* 2004; Pinto *et al.* 2010). The protein encoded by the *CDH13* gene is one of numerous cadherins expressed in the brain, which have been shown to regulate many neural processes (Redies *et al.* 2012) and *CDH13* has been implicated in ADHD through genome-wide association and extended pedigree linkage studies (Lesch *et al.* 2008; Rivero *et al.* 2012). The *RALGDS* and *VAV3* genes are involved in multiple signaling pathways, including nerve growth factor receptor signaling pathway (Ferro and Trabalzini 2010; Keilhoff *et al.* 2012). Therefore, the genes *CDH13*, *RALGDS*, and *VAV3* could represent interesting examples of ASD candidate genes for further follow-up. One of the genes, *DLC1*, contributed to both the “cytoskeleton and contractile fiber” and “small GTPase signaling” pathways. Rare exonic loss in this gene was observed in an ASD case but not in any of the controls. The *DLC1* gene is involved in multiple phenotypes in mouse, including nervous system development (Sabbir *et al.* 2010) and thus is an interesting ASD candidate gene for further study. Examples of some of the genes contributing to the “cytoskeleton and contractile fiber” pathway are *DMD*, *EPB41L2*, *MYH7*, *PGM5*, and *TRIM32*, where we observed rare exonic losses in cases but not in controls. The gene *DMD* has been shown to be associated with ASD in several studies (Wu *et al.* 2005; Hinton *et al.* 2009; Erturk *et al.* 2010). The *TRIM32* gene is suggested to be involved in muscle and nervous system development mouse phenotypes (Kudryashova *et al.* 2009) and deletions in this gene are also reported in cases with attention deficit hyperactivity disorder (Lionel *et al.* 2011). The gene *EPB41L2* binds glutamate (Shen *et al.* 2000) and dopamine receptors (Binda *et al.* 2002) and is also suggested to be involved in mouse reproductive and nervous system development phenotypes (Ivanovic *et al.* 2012). The *PGM5* gene interacts with the *DMD* gene (Wakayama *et al.* 2000; Szklarczyk *et al.* 2011). Thus, we believe this set of ‘Cytoskeleton and Contractile fiber’ pathway genes, which are impacted by ASD-specific variants in the present study, warrant further follow-up.

Whereas much of the focus in identifying ASD genes has been in studying *de novo* events, assessing the role of both autosomal and X-linked rare inherited CNVs also has been fruitful, yielding new susceptibility loci such as *NRXN3* (Vaags *et al.* 2012), *SHANK1* (Sato *et al.* 2012), and the Xp11.2 *PTCHD1-PTCHD1AS* region (Noor *et al.* 2010). However, in taking *de novo* and rare inherited variants together, no one gene accounts for more than 1% of the etiology in ASD highlighting the complex genetic heterogeneity of the disorder. It is, therefore, essential to capture the entire spectrum of genetic variation contributing to ASD risk to account for unexplained heritability. Moreover, given the rarity of some of the putative risk variants identified in this study, it is likely that high resolution genome-wide scans of tens of thousands of ASD cases will be needed to validate and contextualize these findings. The results from the present study will complement ASD genetic risk factors being identified through current whole exome and genome sequencing efforts. The public availability of the new rich resource of rare CNVs uncovered in this study will serve as an important resource for further prioritization of putative ASD risk genes for subsequent genetic and functional characterization.

## Supplementary Material

Supporting Information
