# Supplementary material for: A Discovery Resource of Rare Copy Number Variations in Individuals with Autism Spectrum Disorder
Source: G3 (Bethesda). 2012 Dec 1;2(12):1665–85. doi: 10.1534/g3.112.004689 (PMC3516488; doi:10.1534/g3.112.004689)
Supplement: Supporting Information [file supp_2_12_1665__index.html]

Supporting Information 

# A Discovery Resource of Rare Copy Number Variations in Individuals with Autism Spectrum Disorder

## Supporting Information for Prasad *et al.*, 2012

**Files in this Data Supplement:**

- Supporting Information - Figures S1-S3 and Tables S1-S5 (PDF, 632 KB)
- Figure S1 - Size distribution of the 946 novel and rare CNVs (PDF, 88 KB)
- Figure S2 - (A) Genome browser view of 16p11.2 duplication (B) Genome browser view of 22q11.22-q11.23 duplication region (PDF, 393 KB)
- Figure S3 - Results from ancestry analysis using SNP genotype data (PDF, 234 KB)
- Table S1 - Samples with CNVs larger than 5 Mb in size (PDF, 70 KB)
- Table S3 - ASD cases with deletions in ASD candidate genes (gene list from Betancur et al. 2011) (PDF, 69 KB)
- Table S4 - List of 23 gene-sets enriched for deletions (PDF, 77 KB)
- Table S5 - Genes in the nucleotide metabolism gene-set (PDF, 78 KB)
- Table S2 - List of 1,884 rare CNVs including 946 novel CNVs specific to the ASD dataset (.xls, 248 KB)
