# Supplementary material for: A Discovery Resource of Rare Copy Number Variations in Individuals with Autism Spectrum Disorder
Source: G3 (Bethesda). 2012 Dec 1;2(12):1665–85. doi: 10.1534/g3.112.004689 (PMC3516488; doi:10.1534/g3.112.004689)
Supplement: Supporting Information [file supp_2.12.1665_004689SI.pdf]

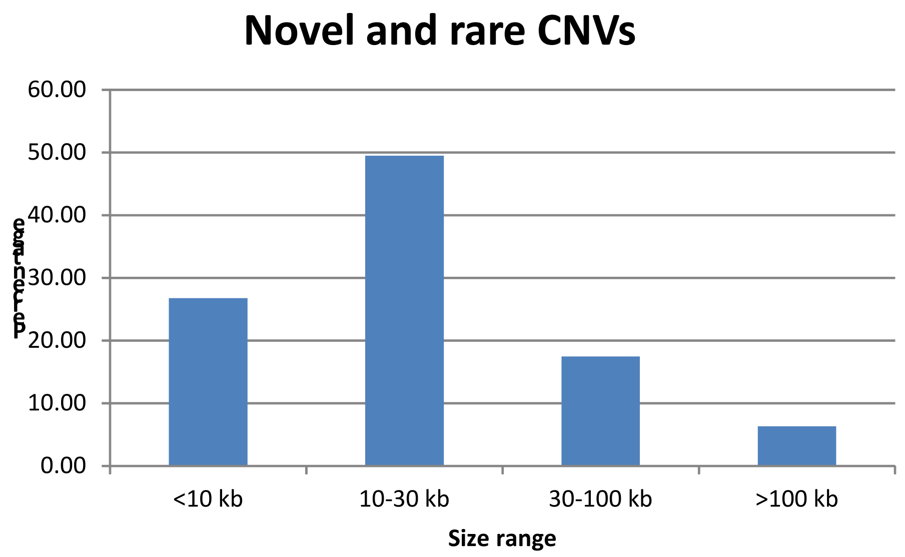

Figure S1 Size distribution of the 946 novel and rare CNVs.

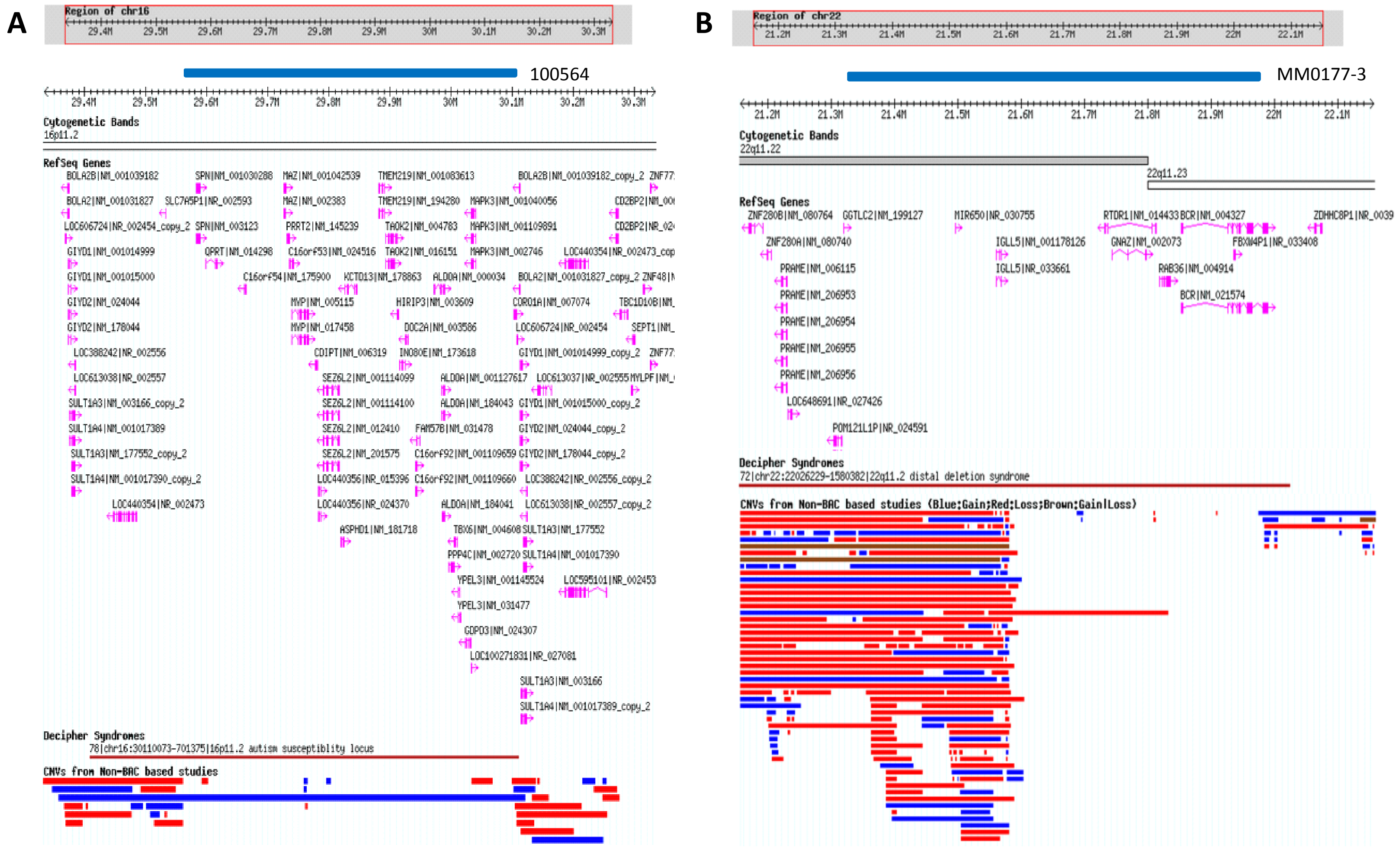

Figure S2 (A) Genome browser view of 16p11.2 duplication (B) Genome browser view of 22q11.22-q11.23 duplication region

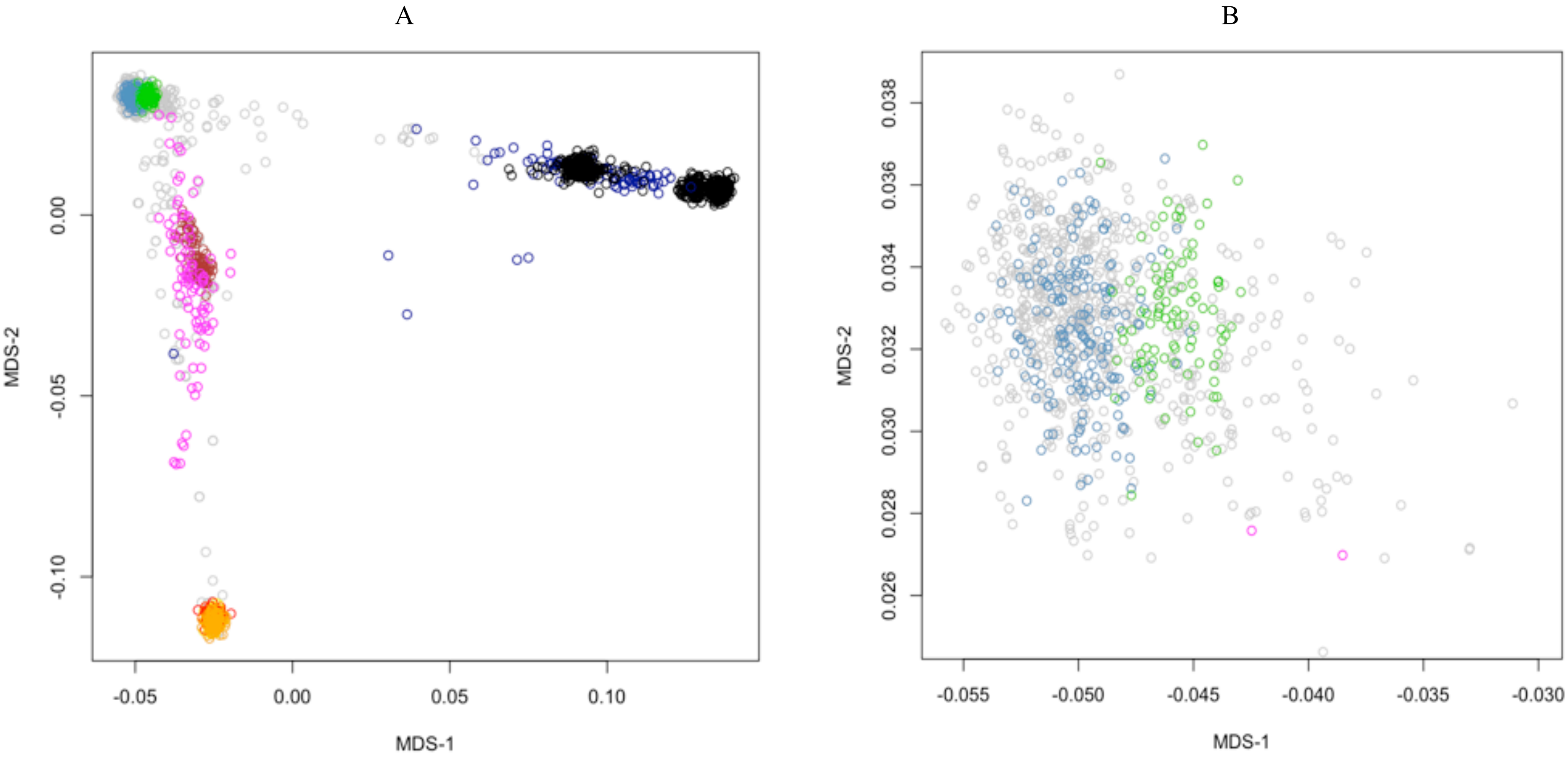
A
MDS-2
MDS-1
0.00
-0.05
-0.10
-0.05
0.00
0.05
0.10
B
MDS-2
MDS-1
0.038
0.036
0.034
0.032
0.030
0.028
0.026
-0.055
-0.050
-0.045
-0.040
-0.035
-0.030

C

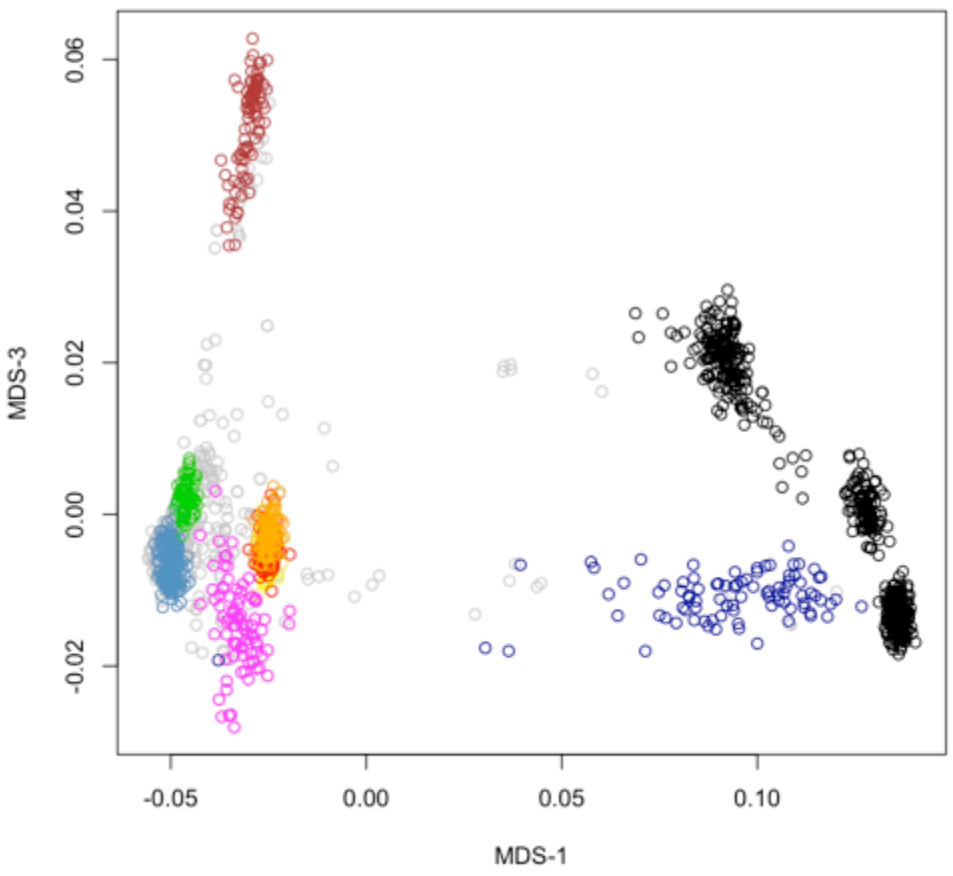

Figure S3 Results from ancestry analysis using SNP genotype data**.** (A) The figure shows the dimensions 1 and 2 of the multidimensional scaling. (B) A zoomed view of the known and putative European group of the dimensions 1 and 2 (C) Plot of dimensions 1 and 3 of the multidimensional scaling.

ASD samples are colored as gray and HapMap3 samples are colored in different colors.

Utah residents with ancestry from northern and western Europe (CEU) – light blue; Tuscany in Italy (TSI) – green; Japanese in Tokyo (JPT) – red; Han Chinese in Beijing (CHB) – yellow; Yoruba in Nigeria (YRI), Masai in Kenya (MKK), Luhya in Kenya (LWK)-black; African ancestry in Southwest USA (ASW)-dark blue; Gujarati Indians in Houston (GIH) – brown; Mexicans (MEX)- magenta

Table S1   Samples with CNVs larger than 5 Mb in size

Sample	Sex	Tissue	Chr	Size (bp)	CNV	Karyotype	Other arrays
119975L	M	L	9p22.1-p21.3	4,998,116	loss	n/a	detected by Affy500K, Marshall *et al.* 2008
139364L	M	L	21q21.2-q21.1	6,260,886	gain	46,XY,dup(21)(q?q?) or der(21)ins(21;21)(q?;q?)	detected by Affy6.0
109332	F	B	18q21.1-q23	31,604,736	gain	46,XX,der(11)t(11;18)(q25;q21.1)	detected by Affy6.0
146451L	F	L	7q21.11 - q36.3	80,056,407	complex (loss/gain)	n/a	Possibly a cell-line artifact, not detected by Affy6.0 (blood DNA was used)
97412	M	B	1q42.3 - q44	13,708,317	gain	n/a	detected by Illumina 1M-single array, Pinto *et al.* 2010
89853L	M	L	21	33,487,618	gain	47, XY + 21 (Down Syndrome)	detected by Illumina 1M-single array, Pinto *et al.* 2010; confirmed by karyotyping
50800L	M	L	7q31.1-q31.31	11,033,516	loss	XY, del(7)(q31)	detected by Affy500K, Marshall *et al.* 2008
85181L	M	L	7q22.2 - q35	38,410,895	gain	46,XY,dup(7)(q22q34)	detected by Affy6.0
60974L	F	L	5p15.33 - p15.2	13,783,361	loss	46,XX,del(5)(p15.1)	detected by Affy500K, Marshall *et al.* 2008
72871L	M	L	3p14.1	5,375,845	loss	t(6;14)(q13;q21)	detected by Affy500K, Marshall *et al.* 2008
165457L	M	L	21	33,580,687	gain	n/a	detected by Affy6.0
165445L	F	L	21	33,580,887	gain	n/a	was not run on any other array, but proband has Down syndrome
60433-L	F	L	7q31.1 - q32.1	15,437,215	loss	XX, del(7)(q31.2q31.3)	detected by Affy500K, Marshall *et al.* 2008
56034	M	B	21	32,875,937	gain	46,XY,+21 (trisomy 21)	was not run on any other array,

							but proband has Down syndrome
59172L	M	L	2p13.3-p25.3	70,640,252	gain	n/a	Possibly a cell-line artifact, not detected by Illumina 1M array, DNA source-cell line for Agilent and blood for Illumina 1M array
60340	F	B	18q21.32 - q23	20,357,135	loss	46, XX, del (18)(q21)	detected by Affy500K, Marshall *et al.* 2008
115733L	M	L	15q11.2 - q13.3	11,634,435	gain	46,XY,trp(15)(q11.2q13)	detected by Affy500K, Marshall *et al.* 2008
82361L	F	L	2p25.3 - p15; Xp22.33 - p22.31	63,366,686; 6,017,794	complex (loss/gain); loss	46,XX,t(11;12)(q23.3;p13.3)	detected by Affy6.0 (same DNA source as Agilent)
57283L	F	L	15q11.1 - q13.3	11,887,780	gain	isodisomy Chr.15	detected by Affy500K, Marshall *et al.* 2008
100569L	F	L	1q21.1-1q21.3	8,315,572	gain	46,XX (17 cells), 46,XX,+ring; Ring chromosome 1	Marshall et al. 2008

Abbreviations: B-blood; L-cell line

Table S2 List of 1,884 rare CNVs including 946 novel CNVs specific to the ASD dataset

Data available for download as an excel file at http://www.g3journal.org/lookup/suppl/doi:10.1534/g3.112.004689/-/DC1.

Table S3 ASD cases with deletions in ASD candidate genes (gene list from Betancur et al. 2011)

GeneID	Symbol	Name	Case counts	Control counts	ASD_%	CT_%	Pvalue
8831	*SYNGAP1*	synaptic Ras GTPase activating protein 1	4	0	2.051	0	0.017308
139411	*PTCHD1*	patched domain containing 1	1	0	0.513	0	0.360958
157680	*VPS13B*	vacuolar protein sorting 13 homolog B (yeast)	1	0	0.513	0	0.360958
1756	*DMD*	dystrophin	1	0	0.513	0	0.360958
1806	*DPYD*	dihydropyrimidine dehydrogenase	1	0	0.513	0	0.360958
22941	*SHANK2*	SH3 and multiple ankyrin repeat domains 2	1	0	0.513	0	0.360958
4763	*NF1*	neurofibromin 1	1	0	0.513	0	0.360958
9378	*NRXN1*	neurexin 1	1	0	0.513	0	0.360958
26047	*CNTNAP2*	contactin associated protein-like 2	0	1	0	0.288	1

Table S4 List of 23 gene-sets enriched for deletions

GsName[a]	GsID[b]	GsSize[c]	Case counts[d]	Control counts[e]	ASD_%[f]	CT_%[g]	p-value[h]	FDR[i]
REACT: Metabolism of nucleotides	REACT:218	77	8	0	4.102564	0	0.000256	0.0784
nucleobase metabolic process	GO:0009112	58	6	0	3.076923	0	0.002063	0.24135
nucleoside metabolic process	GO:0009116	83	6	0	3.076923	0	0.002063	0.24135
KEGG: Drug metabolism - other enzymes	KEGG:00983	52	6	0	3.076923	0	0.002063	0.24135
actin cytoskeleton	GO:0015629	293	18	11	9.230769	3.170029	0.002986	0.23512
structural molecule activity	GO:0005198	606	17	10	8.717949	2.881844	0.003163	0.198033
KEGG: Purine metabolism	KEGG:00230	161	8	2	4.102564	0.576369	0.005467	0.282829
regulation of small GTPase mediated signal transduction	GO:0051056	354	17	11	8.717949	3.170029	0.005476	0.2495
ribonucleoside metabolic process	GO:0009119	58	5	0	2.564103	0	0.005831	0.3022
nucleoside catabolic process	GO:0009164	25	5	0	2.564103	0	0.005831	0.3022
heterocycle catabolic process	GO:0046700	457	16	10	8.205128	2.881844	0.005875	0.279127
nucleobase, nucleoside, nucleotide and nucleic acid catabolic process	GO:0034655	435	15	9	7.692308	2.59366	0.006255	0.241615
nucleobase, nucleoside and nucleotide catabolic process	GO:0034656	435	15	9	7.692308	2.59366	0.006255	0.241615
cellular aromatic compound metabolic process	GO:0006725	193	9	3	4.615385	0.864553	0.006384	0.206153
myofibril	GO:0030016	116	9	3	4.615385	0.864553	0.006384	0.206153
contractile fiber	GO:0043292	123	9	3	4.615385	0.864553	0.006384	0.206153
contractile fiber part	GO:0044449	113	9	3	4.615385	0.864553	0.006384	0.206153
GTPase regulator activity	GO:0030695	454	19	14	9.74359	4.034582	0.007494	0.229522
cell surface	GO:0009986	373	6	1	3.076923	0.288184	0.010077	0.26671
PFAM: Kelch motif	PF01344	68	6	1	3.076923	0.288184	0.010077	0.26671
small GTPase mediated signal transduction	GO:0007264	579	20	16	10.25641	4.610951	0.010406	0.255448
cellular nitrogen compound catabolic process	GO:0044270	461	15	10	7.692308	2.881844	0.010658	0.2462
nucleoside-triphosphatase regulator activity	GO:0060589	466	19	15	9.74359	4.322767	0.011547	0.241191

[a] Name of gene-set, [b] Gene-set ID, [c] Total numbers of genes in a gene-set, [d] Number of ASD cases with one or more CNVs in this gene-set, [e] Number of controls with one or more CNVs in this gene-set, [f] Percentage of ASD cases with at least one gene-set affected by a rare CNV, [g] Percentage of controls with at least one gene-set affected by a rare CNV, [h] Fisher's exact test p-value, [i] False discovery rate.

Table S5 Genes in the nucleotide metabolism gene-set

GeneID	Symbol	Name	Case counts	Control counts	ASD_%	CT_%	Pvalue
2272	*FHIT*	fragile histidine triad gene	2	0	1.026	0	0.130714
1806	*DPYD*	dihydropyrimidine dehydrogenase	1	0	0.513	0	0.360958
272	*AMPD3*	adenosine monophosphate deaminase 3	1	0	0.513	0	0.360958
2766	*GMPR*	guanosine monophosphate reductase	1	0	0.513	0	0.360958
3615	*IMPDH2*	IMP (inosine 5'-monophosphate) dehydrogenase 2	1	0	0.513	0	0.360958
4833	*NME4*	non-metastatic cells 4, protein expressed in	1	0	0.513	0	0.360958
5151	*PDE8A*	phosphodiesterase 8A	1	0	0.513	0	0.360958
51733	*UPB1*	ureidopropionase, beta	1	0	0.513	0	0.360958
5426	*POLE*	polymerase (DNA directed), epsilon	1	0	0.513	0	0.360958
7378	*UPP1*	uridine phosphorylase 1	1	0	0.513	0	0.360958
9154	*SLC28A1*	solute carrier family 28 (sodium-coupled nucleoside transporter), member 1	1	0	0.513	0	0.360958
5137	*PDE1C*	phosphodiesterase 1C, calmodulin-dependent 70kDa	0	1	0	0.288	1
956	*ENTPD3*	ectonucleoside triphosphate diphosphohydrolase 3	0	1	0	0.288	1
