# Supplementary figures and images for: A Discovery Resource of Rare Copy Number Variations in Individuals with Autism Spectrum Disorder
Source: G3 (Bethesda). 2012 Dec 1;2(12):1665–85. doi: 10.1534/g3.112.004689 (PMC3516488; doi:10.1534/g3.112.004689)

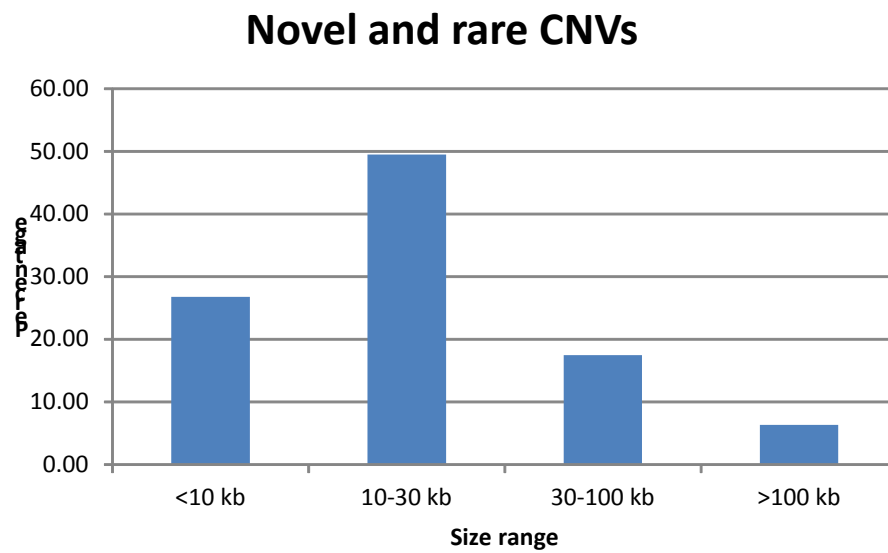

**Figure S1** Size distribution of the 946 novel and rare CNVs.

Supplement: Supporting Information [file supp_2.12.1665_FigureS1.pdf]

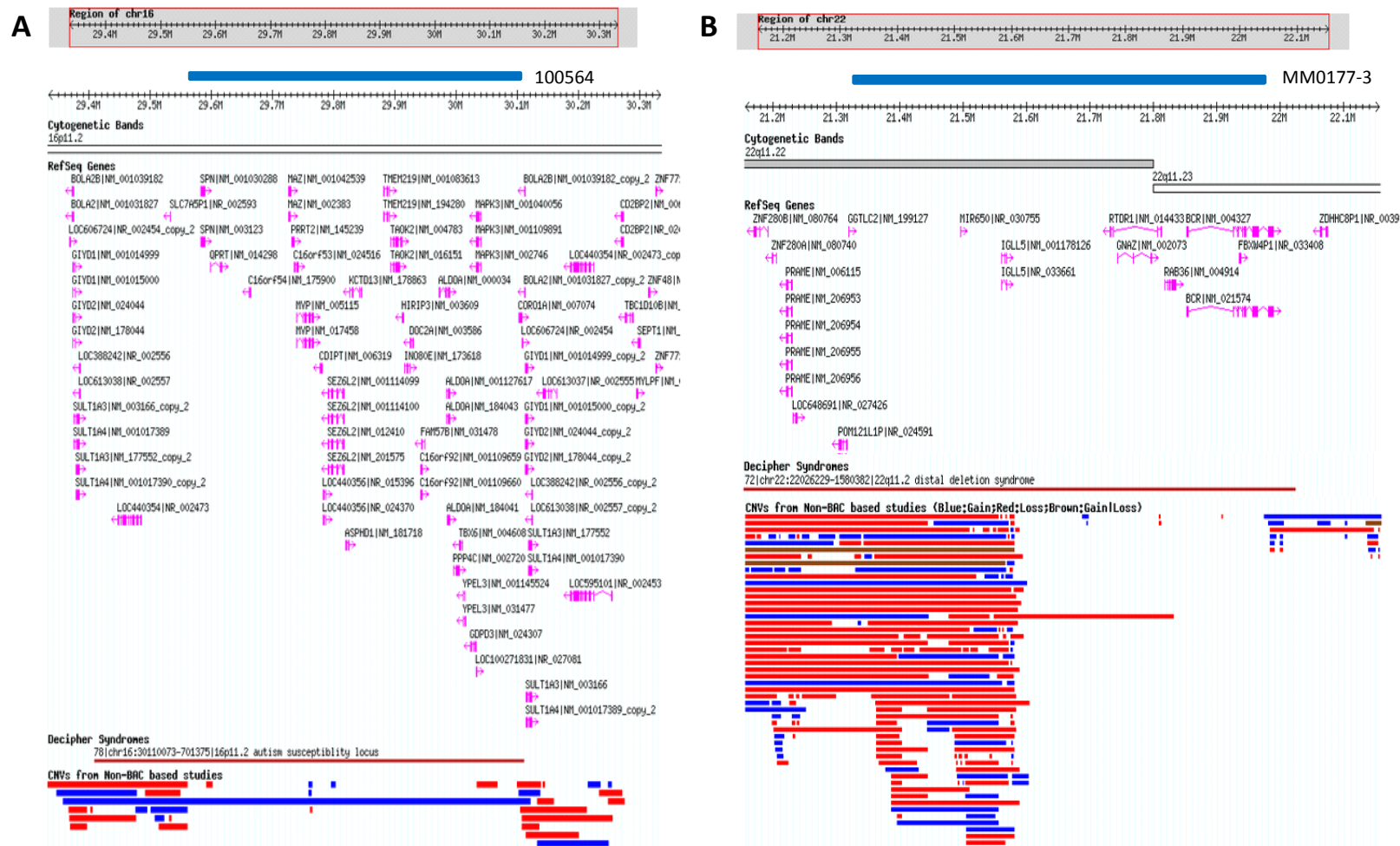

**Figure S2** (A) Genome browser view of 16p11.2 duplication (B) Genome browser view of 22q11.22-q11.23 duplication region

Supplement: Supporting Information [file supp_2.12.1665_FigureS2.pdf]
