# Supplementary material for: A Discovery Resource of Rare Copy Number Variations in Individuals with Autism Spectrum Disorder
Source: G3 (Bethesda). 2012 Dec 1;2(12):1665–85. doi: 10.1534/g3.112.004689 (PMC3516488; doi:10.1534/g3.112.004689)
Supplement: Supporting Information [file supp_2.12.1665_FigureS3.pdf]

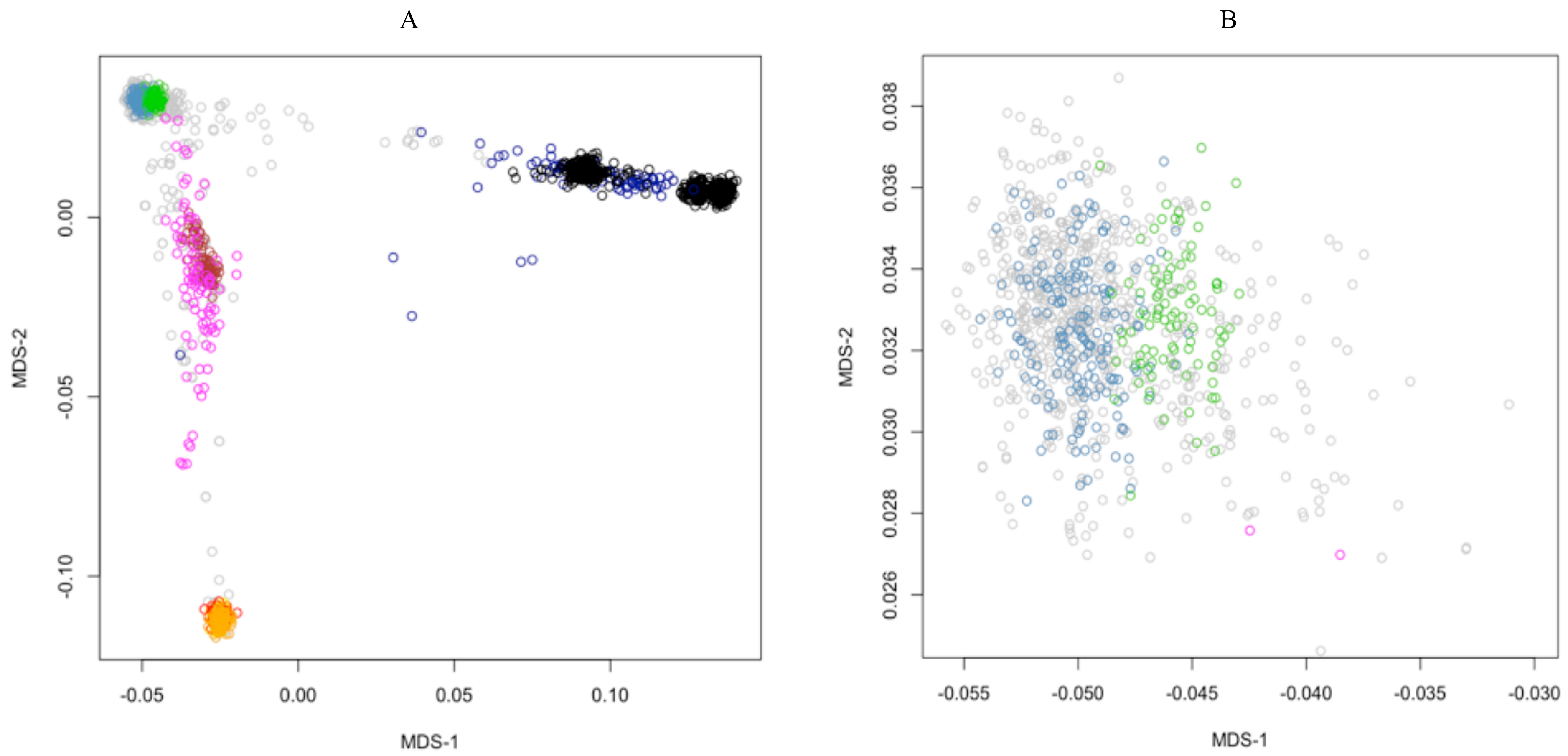

A
B
MDS-2
MDS-1
0.00
-0.05
-0.10
0.05
0.10
0.038
0.036
0.034
0.032
0.030
0.028
0.026
-0.055
-0.050
-0.045
-0.040
-0.035
-0.030

C

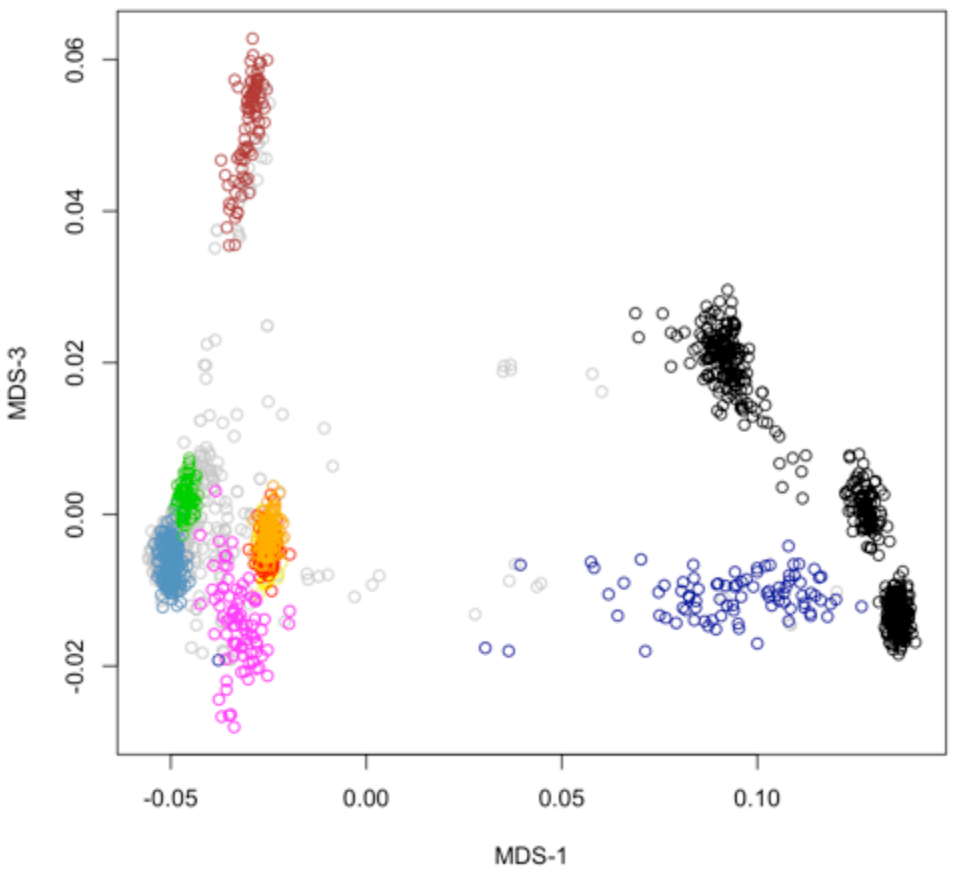

Figure S3 Results from ancestry analysis using SNP genotype data**.** (A) The figure shows the dimensions 1 and 2 of the multidimensional scaling. (B) A zoomed view of the known and putative European group of the dimensions 1 and 2 (C) Plot of dimensions 1 and 3 of the multidimensional scaling.

ASD samples are colored as gray and HapMap3 samples are colored in different colors.

Utah residents with ancestry from northern and western Europe (CEU) – light blue; Tuscany in Italy (TSI) – green; Japanese in Tokyo (JPT) – red; Han Chinese in Beijing (CHB) – yellow; Yoruba in Nigeria (YRI), Masai in Kenya (MKK), Luhya in Kenya (LWK)-black; African ancestry in Southwest USA (ASW)-dark blue; Gujarati Indians in Houston (GIH) – brown; Mexicans (MEX)- magenta
