# Supplementary material for: A Discovery Resource of Rare Copy Number Variations in Individuals with Autism Spectrum Disorder
Source: G3 (Bethesda). 2012 Dec 1;2(12):1665–85. doi: 10.1534/g3.112.004689 (PMC3516488; doi:10.1534/g3.112.004689)
Supplement: Supporting Information [file supp_2.12.1665_TableS1.pdf]

**Table S1   Samples with CNVs larger than 5 Mb in size**

| Sample | Sex | Tissue | Chr | Size (bp) | CNV | Karyotype | Other arrays |
|---|---|---|---|---|---|---|---|
| 119975L | M | L | 9p22.1-p21.3 | 4,998,116 | loss | n/a | detected by Affy500K, Marshall *et al.* 2008 |
| 139364L | M | L | 21q21.2-q21.1 | 6,260,886 | gain | 46,XY,dup(21)(q?q?) or der(21)ins(21;21)(q?;q?) | detected by Affy6.0 |
| 109332 | F | B | 18q21.1-q23 | 31,604,736 | gain | 46,XX,der(11)t(11;18)(q25;q21.1) | detected by Affy6.0 |
| 146451L | F | L | 7q21.11 - q36.3 | 80,056,407 | complex (loss/gain) | n/a | Possibly a cell-line artifact, not detected by Affy6.0 (blood DNA was used) |
| 97412 | M | B | 1q42.3 - q44 | 13,708,317 | gain | n/a | detected by Illumina 1M-single array, Pinto *et al.* 2010 |
| 89853L | M | L | 21 | 33,487,618 | gain | 47, XY + 21 (Down Syndrome) | detected by Illumina 1M-single array, Pinto *et al.* 2010; confirmed by karyotyping |
| 50800L | M | L | 7q31.1-q31.31 | 11,033,516 | loss | XY, del(7)(q31) | detected by Affy500K, Marshall *et al.* 2008 |
| 85181L | M | L | 7q22.2 - q35 | 38,410,895 | gain | 46,XY,dup(7)(q22q34) | detected by Affy6.0 |
| 60974L | F | L | 5p15.33 - p15.2 | 13,783,361 | loss | 46,XX,del(5)(p15.1) | detected by Affy500K, Marshall *et al.* 2008 |
| 72871L | M | L | 3p14.1 | 5,375,845 | loss | t(6;14)(q13;q21) | detected by Affy500K, Marshall *et al.* 2008 |
| 165457L | M | L | 21 | 33,580,687 | gain | n/a | detected by Affy6.0 |
| 165445L | F | L | 21 | 33,580,887 | gain | n/a | was not run on any other array, but proband has Down syndrome |
| 60433-L | F | L | 7q31.1 - q32.1 | 15,437,215 | loss | XX, del(7)(q31.2q31.3) | detected by Affy500K, Marshall *et al.* 2008 |
| 56034 | M | B | 21 | 32,875,937 | gain | 46,XY,+21 (trisomy 21) | was not run on any other array, |

| | | | | | | | |
|---|---|---|---|---|---|---|---|
| | | | | | | | but proband has Down syndrome |
| 59172L | M | L | 2p13.3-p25.3 | 70,640,252 | gain | n/a | Possibly a cell-line artifact, not detected by Illumina 1M array, DNA source-cell line for Agilent and blood for Illumina 1M array |
| 60340 | F | B | 18q21.32 - q23 | 20,357,135 | loss | 46, XX, del (18)(q21) | detected by Affy500K, Marshall *et al.* 2008 |
| 115733L | M | L | 15q11.2 - q13.3 | 11,634,435 | gain | 46,XY,trp(15)(q11.2q13) | detected by Affy500K, Marshall *et al.* 2008 |
| 82361L | F | L | 2p25.3 - p15; Xp22.33 - p22.31 | 63,366,686; 6,017,794 | complex (loss/gain); loss | 46,XX,t(11;12)(q23.3;p13.3) | detected by Affy6.0 (same DNA source as Agilent) |
| 57283L | F | L | 15q11.1 - q13.3 | 11,887,780 | gain | isodisomy Chr.15 | detected by Affy500K, Marshall *et al.* 2008 |
| 100569L | F | L | 1q21.1-1q21.3 | 8,315,572 | gain | 46,XX (17 cells), 46,XX,+ring; Ring chromosome 1 | Marshall et al. 2008 |

Abbreviations: B-blood; L-cell line
