# Supplementary material for: A Discovery Resource of Rare Copy Number Variations in Individuals with Autism Spectrum Disorder
Source: G3 (Bethesda). 2012 Dec 1;2(12):1665–85. doi: 10.1534/g3.112.004689 (PMC3516488; doi:10.1534/g3.112.004689)
Supplement: Supporting Information [file supp_2.12.1665_TableS3.pdf]

Table S3 ASD cases with deletions in ASD candidate genes (gene list from Betancur et al. 2011)

GeneID	Symbol	Name	Case counts	Control counts	ASD_%	CT_%	Pvalue
8831	*SYNGAP1*	synaptic Ras GTPase activating protein 1	4	0	2.051	0	0.017308
139411	*PTCHD1*	patched domain containing 1	1	0	0.513	0	0.360958
157680	*VPS13B*	vacuolar protein sorting 13 homolog B (yeast)	1	0	0.513	0	0.360958
1756	*DMD*	dystrophin	1	0	0.513	0	0.360958
1806	*DPYD*	dihydropyrimidine dehydrogenase	1	0	0.513	0	0.360958
22941	*SHANK2*	SH3 and multiple ankyrin repeat domains 2	1	0	0.513	0	0.360958
4763	*NF1*	neurofibromin 1	1	0	0.513	0	0.360958
9378	*NRXN1*	neurexin 1	1	0	0.513	0	0.360958
26047	*CNTNAP2*	contactin associated protein-like 2	0	1	0	0.288	1
