# Supplementary material for: A Discovery Resource of Rare Copy Number Variations in Individuals with Autism Spectrum Disorder
Source: G3 (Bethesda). 2012 Dec 1;2(12):1665–85. doi: 10.1534/g3.112.004689 (PMC3516488; doi:10.1534/g3.112.004689)
Supplement: Supporting Information [file supp_2.12.1665_TableS4.pdf]

Table S4 List of 23 gene-sets enriched for deletions

GsName[a]	GsID[b]	GsSize[c]	Case counts[d]	Control counts[e]	ASD_%[f]	CT_%[g]	p-value[h]	FDR[i]
REACT: Metabolism of nucleotides	REACT:218	77	8	0	4.102564	0	0.000256	0.0784
nucleobase metabolic process	GO:0009112	58	6	0	3.076923	0	0.002063	0.24135
nucleoside metabolic process	GO:0009116	83	6	0	3.076923	0	0.002063	0.24135
KEGG: Drug metabolism - other enzymes	KEGG:00983	52	6	0	3.076923	0	0.002063	0.24135
actin cytoskeleton	GO:0015629	293	18	11	9.230769	3.170029	0.002986	0.23512
structural molecule activity	GO:0005198	606	17	10	8.717949	2.881844	0.003163	0.198033
KEGG: Purine metabolism	KEGG:00230	161	8	2	4.102564	0.576369	0.005467	0.282829
regulation of small GTPase mediated signal transduction	GO:0051056	354	17	11	8.717949	3.170029	0.005476	0.2495
ribonucleoside metabolic process	GO:0009119	58	5	0	2.564103	0	0.005831	0.3022
nucleoside catabolic process	GO:0009164	25	5	0	2.564103	0	0.005831	0.3022
heterocycle catabolic process	GO:0046700	457	16	10	8.205128	2.881844	0.005875	0.279127
nucleobase, nucleoside, nucleotide and nucleic acid catabolic process	GO:0034655	435	15	9	7.692308	2.59366	0.006255	0.241615
nucleobase, nucleoside and nucleotide catabolic process	GO:0034656	435	15	9	7.692308	2.59366	0.006255	0.241615
cellular aromatic compound metabolic process	GO:0006725	193	9	3	4.615385	0.864553	0.006384	0.206153
myofibril	GO:0030016	116	9	3	4.615385	0.864553	0.006384	0.206153
contractile fiber	GO:0043292	123	9	3	4.615385	0.864553	0.006384	0.206153
contractile fiber part	GO:0044449	113	9	3	4.615385	0.864553	0.006384	0.206153
GTPase regulator activity	GO:0030695	454	19	14	9.74359	4.034582	0.007494	0.229522
cell surface	GO:0009986	373	6	1	3.076923	0.288184	0.010077	0.26671
PFAM: Kelch motif	PF01344	68	6	1	3.076923	0.288184	0.010077	0.26671
small GTPase mediated signal transduction	GO:0007264	579	20	16	10.25641	4.610951	0.010406	0.255448
cellular nitrogen compound catabolic process	GO:0044270	461	15	10	7.692308	2.881844	0.010658	0.2462
nucleoside-triphosphatase regulator activity	GO:0060589	466	19	15	9.74359	4.322767	0.011547	0.241191

[a] Name of gene-set, [b] Gene-set ID, [c] Total numbers of genes in a gene-set, [d] Number of ASD cases with one or more CNVs in this gene-set, [e] Number of controls with one or more CNVs in this gene-set, [f] Percentage of ASD cases with at least one gene-set affected by a rare CNV, [g] Percentage of controls with at least one gene-set affected by a rare CNV, [h] Fisher's exact test p-value, [i] False discovery rate.
