# Supplementary material for: A Discovery Resource of Rare Copy Number Variations in Individuals with Autism Spectrum Disorder
Source: G3 (Bethesda). 2012 Dec 1;2(12):1665–85. doi: 10.1534/g3.112.004689 (PMC3516488; doi:10.1534/g3.112.004689)
Supplement: Supporting Information [file supp_2.12.1665_TableS5.pdf]

Table S5 Genes in the nucleotide metabolism gene-set

GeneID	Symbol	Name	Case counts	Control counts	ASD_%	CT_%	Pvalue
2272	*FHIT*	fragile histidine triad gene	2	0	1.026	0	0.130714
1806	*DPYD*	dihydropyrimidine dehydrogenase	1	0	0.513	0	0.360958
272	*AMPD3*	adenosine monophosphate deaminase 3	1	0	0.513	0	0.360958
2766	*GMPR*	guanosine monophosphate reductase	1	0	0.513	0	0.360958
3615	*IMPDH2*	IMP (inosine 5'-monophosphate) dehydrogenase 2	1	0	0.513	0	0.360958
4833	*NME4*	non-metastatic cells 4, protein expressed in	1	0	0.513	0	0.360958
5151	*PDE8A*	phosphodiesterase 8A	1	0	0.513	0	0.360958
51733	*UPB1*	ureidopropionase, beta	1	0	0.513	0	0.360958
5426	*POLE*	polymerase (DNA directed), epsilon	1	0	0.513	0	0.360958
7378	*UPP1*	uridine phosphorylase 1	1	0	0.513	0	0.360958
9154	*SLC28A1*	solute carrier family 28 (sodium-coupled nucleoside transporter), member 1	1	0	0.513	0	0.360958
5137	*PDE1C*	phosphodiesterase 1C, calmodulin-dependent 70kDa	0	1	0	0.288	1
956	*ENTPD3*	ectonucleoside triphosphate diphosphohydrolase 3	0	1	0	0.288	1
